# Identification and Functional Characterization of Tomato CircRNAs Derived from Genes Involved in Fruit Pigment Accumulation

**DOI:** 10.1038/s41598-017-08806-0

**Published:** 2017-08-17

**Authors:** Jinjuan Tan, Zhongjing Zhou, Yujie Niu, Xiaoyong Sun, Zhiping Deng

**Affiliations:** 1 State Key Laboratory Breeding Base for Zhejiang Sustainable Pest and Disease Control, Institute of Virology and Biotechnology, Zhejiang Academy of Agricultural Sciences, Hangzhou, 310021 China; 2 Agricultural Big-Data Research Center, College of Information Science And Engineering, Shandong Agricultural University, Taian, Shandong 271018 China

## Abstract

CircRNAs, a class of widespread circular RNAs produced from precursor mRNA back-splicing, have been implicated in regulation of gene expression in eukaryotes, but their biological functions in plants have not yet been elucidated. By deep sequencing of rRNA-removed and RNase R-digested RNA samples we have identified several thousands of putative back-splicing sites in tomato fruit (*Solanum lycopersicum*) and show that the abundance of some of these circRNAs derived from fruit pigment biosynthesis genes are regulated by fruit ripening. Herein, we overexpressed a circRNA derived from *Phytoene Synthase* 1 (*PSY1*) in tomato ‘Ailsa Craig’ and microTom. The *PSY1* mRNA abundance, the lycopene and β-carotene accumulation were decreased significantly in the transgenic tomato fruits, likely due to the continuous highly expressed circRNAs and/or the low abundant linear RNAs generated from the overexpression vector. Besides, overexpression of a circRNA derived from *Phytoene Desaturase* (*PDS*) showed similar results. Our results provide biological insights into plant circRNAs.

## Introduction

CircRNAs, a class of circular RNAs in eukaryotes, are derived from precursor mRNA back-splicing^1^. Although circRNAs have been identified more than 20 years ago^2–4^, they had been considered to be produced from aberrant splicing and their existence and functional potential were both underestimated. Nowadays, with the development of next-generation sequencing and bioinformatics, circRNAs have been identified in various eukaryotic species^5–9^. Most of the identified circRNAs are expressed at low levels, indicating the possibility that the majority of circRNAs might be splicing byproducts with little functional potential^8, 10–12^. However, many circRNAs are more abundant than their linear counterparts^5–8, 13^, suggesting the potential functional significance of these circular RNA molecules. Recent studies revealed that circRNAs are more stable than linear mRNAs, and most of them are cytoplasmic^6, 7, 13^. In addition, the circularization of circRNAs are conserved among species, and the expression of circRNAs are often cell, tissue and developmental stage-specific^7, 8, 13–15^. Recent studies have revealed that circRNAs may play roles in gene expression regulation, although the function of most circRNAs remain largely unknown^16–18^.

The biogenesis of circRNAs is considered to be regulated by both *cis*-elements and *trans*-acting factors^16, 18^. Complementary sequences or inverted repeats in the introns flanking the back-splice site could promote exon circularization by pairing to form hairpin structures^10, 13, 19, 20^, and multiple circRNAs may be produced from one single gene due to different sequence pairing, which is referred as to alternative circularization^10^. However, there are also circRNAs produced from exons without being bracketed by complementary sequences^21–23^, indicating that other *cis*-elements may account for the circularization, such as sequences recognized by RNA-binding proteins (RBPs)^24–26^. Exon circularization may occur during transcription^20, 24^ or post-transcriptionally^19, 27^, with *trans*-acting factors, such as the splicing factor Muscleblind (MBL)^24^ and Quaking (QKI)^25^. The circRNAs have been shown to regulate gene expression by acting as miRNA sponges^7, 28, 29^. However, miRNA inhibition may not be a general function of circRNAs since very few of them contain multiple binding sites for specific miRNAs^12, 22, 30, 31^. The biogenesis of circMbl, a circRNA in *Drosophila*, were demonstrated to compete with pre-mRNA splicing by binding and sequestering the splicing factor MBL indicating role of circRNAs in gene splicing^24^. A subtype of circRNAs with retained intron(s) (termed as exon-intron circRNAs or EIciRNAs), are mainly localized in the nucleus and revealed to promote the transcription of their parent genes providing evidence for direct regulation of transcription by circRNAs^32^. Moreover, recent studies demonstrated that circRNAs can be translated *in vitro* and *in vivo* ^33–36^.

In recent years, a large number of circRNAs have been identified and characterized in various eukaryotes, especially in animals, but much less has been known in plants, except the identification and validation of circRNAs in Arabidopsis, rice (*Oryza sativa* L.), tomato (*Solanum lycopersicum* L.), and barley (*Hordeum vulgare* L.)^9, 22, 37–40^. Tomato fruit has been served as the model system for studying fruit ripening, and has been extensively studied at the physiological, biochemical, molecular and genetic levels. The biosynthetic pathway of carotenoids in tomato fruit is well elucidated, but its regulation is complex and still needs to be clarified. Herein, by deep sequencing, we performed genome-wide identification of circRNAs in tomato fruits at different ripening stages, and analyzed the function significance of one special circRNA derived from *Phytoene Synthase 1 (PSY1)*, a key gene in carotenoid biosynthesis.

## Results

### Bioinformatic detection and molecular validation of back-splice sites in ripening tomato fruits

**Figure 1:**
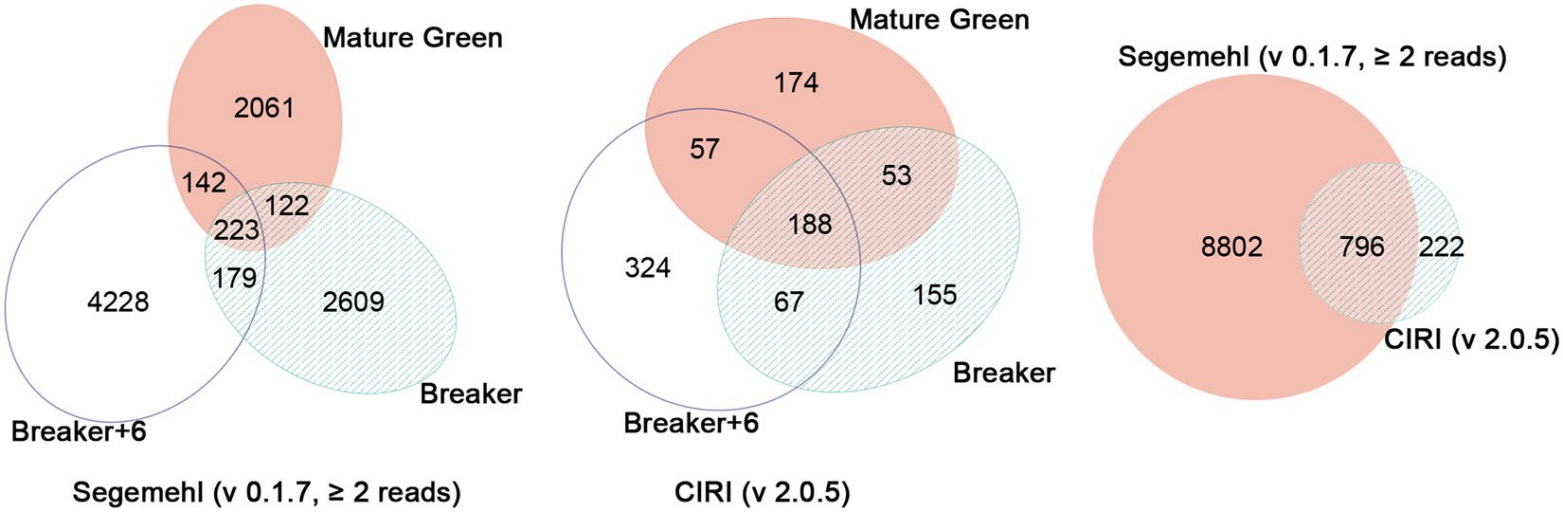
Distribution of circular RNAs among fruits at different ripening stages.

**Figure 2:**
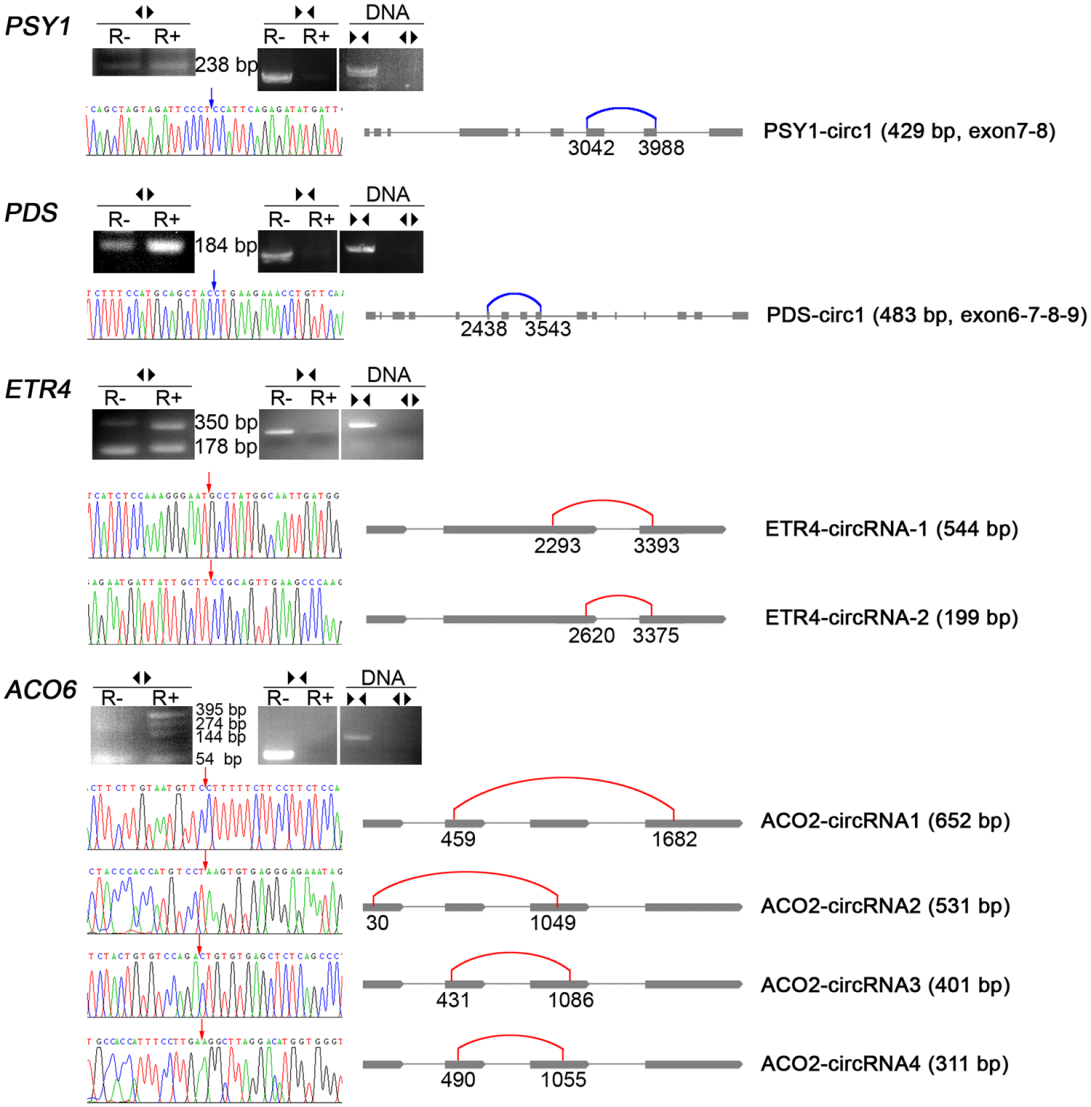
Canonical and non-canonical circRNAs validated by RNase R resistance and sequencing. RNase R resistance analysis. After DNase I treatment, total RNA from tomato fruit pericarp was digested with (R+, one hour at 37 °C) or without (R−, use water instead) RNase R. Both R+ and R− were reverse transcribed to cDNA using random primers. Divergent primers (◀▶) and convergent primers (▶◀) were used to identify circRNAs and linear RNAs respectively. Genomic DNA was also used as template to make certain that the site is not existent in genome. Sanger sequencing of divergent primers-amplified products were showed. The blue arrows and blue curves indicate canonical back-splice sites, while the red arrows and red curves indicate non-canonical back-splice sites.

To obtain circRNA sites in ripening fruits, we deep-sequenced rRNA-removed and RNase R-digested RNA samples of tomato fruits (Ailsa Craig) at mature green, breaker, and breaker +6 stages with two biological duplicates, and obtained a total of 266 million paired-end reads. The potential back-splice junctions were extracted with Segemehl (v 0.1.7) software^41^, and totally 9598 unique potential back-splice sites (≥2 reads) were identified. Although a large number of putative back-splice sites were obtained, the data showed low reproducibility, and only 223 putative back-splice sites were shared among samples (Table 1 and Fig. 1). We also analyzed the deep sequencing data with another tool CIRI (v 2.0.5)^42^, which filters out back-splice junctions without GU/AG splicing signals, and obtained 1018 unique putative circRNAs (with the default setting). The number of overlapped back-splice junctions between the two prediction algorithms is 796 (Fig. 1). The back-splice sites identified by both algorithms are listed in Supplementary Table 1. The circular RNAs within coding regions obtained by Segemehl prediction represented exonic circRNAs with back-splice sites derived from known splice sites (mostly the U2 splice signals (GU/AG), canonical sites)^43^, from unannotated splice sites (non-canonical sites), and intronic circular RNAs (ciRNAs). Validation of circRNAs derived from 16 genes associated with ripening by RNase R resistance and sequencing identified 16 canonical and 37 non-canonical circRNAs (Supplementary Table 2 and Fig. 2). We found that alternative circularization existed extensively in tomato fruits, including alternative back-splicing circularization (different back-splice sites originated from the same gene) and alternative splicing circularization (different transcripts with the same back-splice site) (Supplementary Fig. 1).

**Table 1:** Yield of deep sequencing and putative back-splice sites detected.

Samples	Yield		Segemehl (v 0.1.7, ≥2 reads)		CIRI (v 2.0.5, the default setting)	
	Mbases	#Reads	# circRNAs	Intersection	# circRNAs	Intersection
Mature green-1	4424	43807106	1464	161	271	150
Mature green-2	4701	46551038	1245		351	
Breaker-1	4770	47227328	1762	163	286	155
Breaker-2	4446	44017308	1534		332	
Breaker +6-1	4241	41987128	2427	242	425	180
Breaker +6-2	4291	42494150	2587		391	
Total	26873	266084058	9598		1018

### Expression profiling of circRNAs in tomato fruits

**Figure 3:**
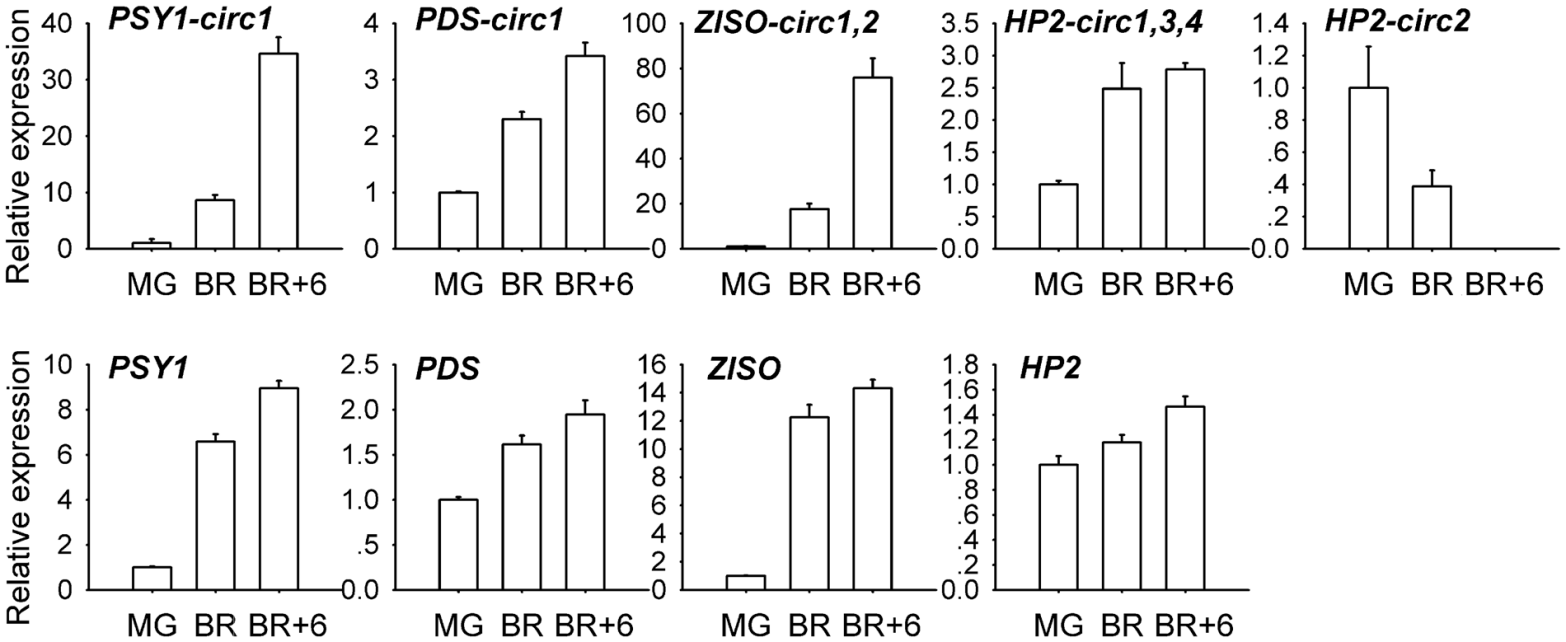
Expression profiling of a few circRNAs and their parent genes in Ailsa Craig fruits at different ripening stages. The data were normalized to *Actin* (NM_001321306.1) and presented as the means ± SD (Standard Deviation, n = 3). MG, mature green. BR, breaker.

The number of putative back-splice sites seems increased during ripening process (Table 1 and Fig. 1), suggesting potential relationship between circRNAs and fruit ripening. To validate this, we characterized the expression of a few canonical circRNAs derived from genes associated with fruit color, including those from *PSY1*, *Phytoene Desaturase* (*PDS)*, *15-cis-zeta-carotene isomerase* (*ZISO*) and *DE-ETIOLATED 1* (*DET1* or *HP2*), and found that most of them were up-regulated during fruit ripening and exhibited expression profiles similar to their parent genes (Fig. 3). The opposite expression trend of circRNAs derived from *HP2* was likely due to alternative and competitive circularization. We also characterized the expression of these circRNAs in an ethylene-insensitive *Nr* (*Never Ripe*) mutant, and found that all these circRNAs were up-regulated during ripening, even though some of their parent genes were down-regulated at breaker +6 stage (Supplementary Fig. 2). These results suggest potential functional significance of some circRNAs in fruit ripening. Herein we focused on circRNAs generated from *PSY1*, a key gene involved in carotenoid biosynthesis. By RNA-seq, we detected all the three classes of circular RNAs (Supplementary Table 3), indicating that alternative circularization occurred on this gene. It seems that the abundance of *PSY1* circRNAs increased during ripening (Supplementary Table 3), especially the three canonical circRNAs (Supplementary Fig. 3), suggesting that *PSY1* circRNAs may be involved in fruit ripening.

### Overexpression of PSY1-circ1 in tomato Ailsa Craig and microTom

**Figure 4:**
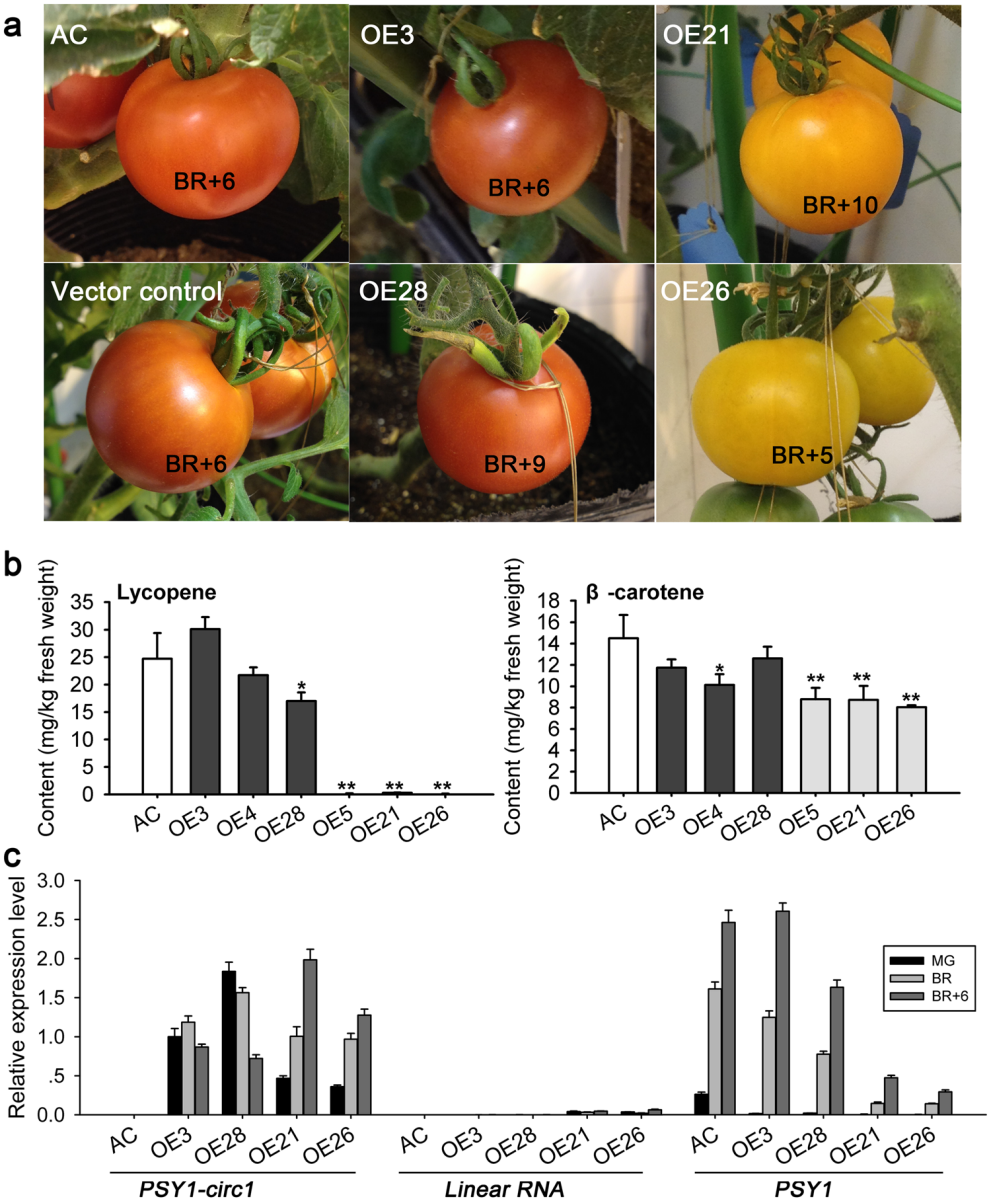
Overexpression of PSY1-circ1 in Ailsa Craig. (**a**) Fruits of different PSY1-circ1 overexpression tomato lines. OE3-OE28 stand for transgenic lines. Vector control stands for Ailsa Craig (AC) plants transformed with the empty vector pCAMBIA1301. (**b**) Lycopene and β-carotene contents in transgenic tomato fruits at breaker +10 stage. White, black and gray bars stand for control, transgenic lines with red fruits and yellow fruits respectively. The data with error bars were expressed as mean ± SEM (Standard Error of the Mean, n = 4), asterisks indicate statistically significant difference (with respect to control; *p value < 0.05; **p value < 0.01; two-way ANOVA followed by LSD (Least Significant Difference) test). (**c**) Expression profile of PSY1-circ1, linear RNAs generated by the overexpression vector, and *PSY1* gene in transgenic tomato fruits at different ripening stages. The data are normalized to *Actin* and presented as means ± SD (n = 5).

To gain insight into the potential function of plant circRNAs in fruit ripening, we overexpressed a canonical circRNA derived from *PSY1* gene (termed as PSY1-circ1) (Fig. 2) in tomato cvs. Ailsa Craig and microTom. We constructed the overexpression vector (using the CaMV 35S promoter) by introducing part of the flanking intron and inserting it into the other side of the exons in an opposite orientation (Supplementary Fig. 4)^28^, as it was shown that the pairing of complementary reverse sequence could promote circRNA biogenesis^10^. With quantitative real-time PCR and sequencing analysis, we found that PSY1-circ1 was highly expressed in the transgenic tomato fruits at different ripening stages (Fig. 4c and Supplementary Fig. 5c), indicating the high efficiency of circRNA overexpression system. To check whether the overexpression vector could generate undesired circular RNAs or large amounts of linear RNAs as shown in a previous report^22^, we used divergent and convergent primers to amplify the potential fragments, and the results indicate that the expression vector could produce the exact circRNA PSY1-circ1 with higher abundance, and also produce linear RNAs with much lower abundance (Supplementary Fig. 6 and Fig. 4c). In the overexpression vector, the endogenous non-complementary flanking sequences (281 bp upstream and 139 bp downstream) were retained (Supplementary Fig. 4), therefore the endogenous sequences flanking the back-splice site might contribute to the precision and high level of back-splicing in plants.

### Phenotype of PSY1-circ1 overexpressed tomato

The PSY1-circ1 overexpression Ailsa Craig plants showed different phenotypes, in which eight lines produced red fruits, while the other seven lines yielded yellow fruits (Fig. 4a and Supplementary Table 4). Similar to the transgenic Ailsa Craig plants, some transgenic microTom lines produced red fruits (24 lines), while the others produced yellow fruits (6 lines) (Supplementary Fig. 5a and Supplementary Table 4). In agreement with the fruit color, both the lycopene and β-carotene contents in the yellow transgenic fruits were decreased significantly, and their contents in some red transgenic fruits were slightly decreased (Fig. 4b and Supplementary Fig. 5b). The decrease of the β-carotene content was slighter than that of the lycopene content, and this result may be explained by the fact that β-carotene accumulates prior to ripening initiation^44^. Although with different fruit color, all the transgenic tomatoes had high expression levels of PSY1-circ1 (Fig. 4c and Supplementary Fig. 5c). We planted hygromycin-resistant T1 generation of a few transgenic microTom lines, and progenies from the yellow T0 lines produced mostly yellow fruits, and progenies from the red T0 lines produced mostly red fruits (Supplementary Table 4), indicating relative stable inheritance of the fruit color phenotype. Similar to T0 generation plants, both T1 generation plants with red and yellow fruits had high expression levels of PSY1-circ1 (Supplementary Fig. 7). In addition, the petals of some transgenic plants bearing yellow fruits (both Ailsa Craig and microTom) were white (Supplementary Fig. 8).

### Expression of PSY1-circ1 and *PSY1* in transgenic tomato fruits

In agreement with the fruit color phenotype, the expression of *PSY1* in the ripening fruits of transgenic Ailsa Craig or microTom plants bearing yellow fruits, was down-regulated, and *PSY1* expression in the red transgenic fruits were higher than in the yellow ones (Fig. 4c and Supplementary Fig. 5c), since *PSY1* is a key gene in lycopene biogenesis. With regard to PSY1-circ1, we found that its expression showed different pattern between the red and yellow transgenic fruits. The expression of PSY1-circ1 in the yellow transgenic Ailsa Craig fruits showed an uptrend from the mature green to the breaker stage, then to the breaker +6 stage, and its expression in the yellow transgenic fruits was more abundant than that in the red ones at the breaker +6 stage. On the contrary, the PSY1-circ1 expression in the red transgenic fruits decreased from the breaker stage to the breaker +6 stage (Fig. 4c). Similar results were obtained in the PSY1-circ1 transgenic microTom fruits (Supplementary Fig. 5c). These results suggest that the continuous high expression of PSY-circ1 may play roles in inhibiting its parent mRNA accumulation. In addition, we found that the linear RNAs generated from the overexpression vector, though with low abundance, were more abundant in the yellow transgenic fruits than in the red ones (Fig. 4c), suggesting the possibility of co-suppression between the linear RNAs and the endogenous mRNAs.

### Overexpression of PDS-circ1 in microTom

**Figure 5:**
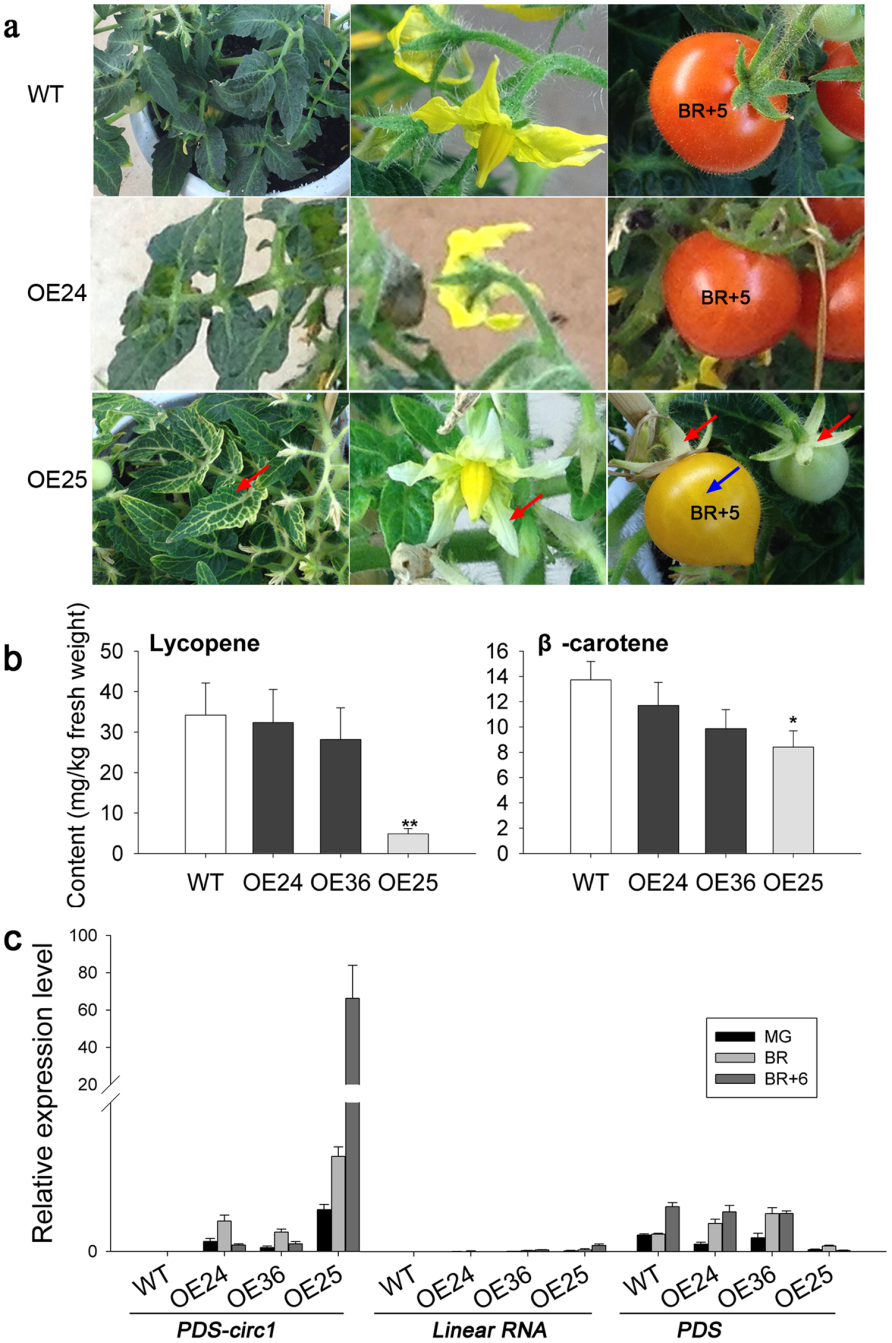
Overexpression of PDS-circ1 in microTom. (**a**) Leaves, flowers and fruits of different PDS-circ1 overexpression lines and the control (WT), red arrows indicate photo-bleaching of leaves, petals, and sepals, blue arrow indicates yellow fruit. (**b**) Lycopene and β-carotene contents in transgenic tomato fruits at breaker +10 stage. White, black and gray bars stand for control, transgenic lines with red fruits and yellow fruits respectively. The data with error bars are expressed as mean ± SEM (n = 5), asterisks indicate statistically significant difference (with respect to control; *p value < 0.05; **p value < 0.01; two-way ANOVA followed by LSD test). (**c**) Expression profile of PDS-circ1, linear RNAs generated by the overexpression vector, and *PDS* gene in transgenic tomato fruits at different ripening stages. The data were normalized to *Actin* and presented as means ± SD (n = 5). OE24-OE36 stand for different transgenic lines.

To examine whether other exonic canonical circRNAs play roles in fruit development and ripening, we overexpressed another circRNA derived from *PDS*, termed as PDS-circ1 (Fig. 2), in microTom in the same way as PSY1-circ1. Although most transgenic plants had no obvious phenotype, a few plants showed photobleached leaves, and a transgenic line (OE25) produced yellow fruits with photo-bleaching on the leaves, petals, and sepals (Fig. 5a). We found that the *PDS* expression level and the lycopene and β-carotene contents in the fruits of OE25 were decreased significantly (Fig. 5b,c), which is in agreement with the fruit color phenotype, since *PDS* is another critical gene in carotenoid biosynthesis. Besides, photo-bleaching is a typical phenotype of *PDS* silencing^45^. The results indicate that PDS-circ1 was highly expressed in all the transgenic tomato fruits, and its expression in OE25 fruits was increased dramatically after the breaker stage, and was even higher at the breaker +6 stage, while its expression in the fruits of the other transgenic lines, was much lower at the breaker +6 stage (Fig. 5c). Furthermore, the linear RNAs generated from the overexpression vector were expressed at low levels in the fruits of all the transgenic lines, and its expression in OE25 fruits was relatively higher than in the fruits of other transgenic lines (Fig. 5c). Therefore, these results suggest that tomato plants with the highest expression of PDS-circ1 showed inhibition of *PDS* expression in leaves and fruits.

## Discussion

Most of the putative circRNAs we identified were expressed at low levels, which is consistent with recent reports^8, 10–12^, and suggesting little functional potential of most circRNAs. It seems that there is potential relationship between circRNAs and fruit ripening since the total number of putative circRNAs was increased and a few circRNAs we validated were upregulated similar to their parent genes during ripening process. As reported in other plants^37^, the positive correlation of expression pattern between some circRNAs and their parent genes indicating that their biogenesis might share similar mechanism. However, in *Nr* mutant, PSY1-circ1 and PDS-circ1 were upregulated after the breaker stage while their parent genes were downregulated. Therefore, the relationship of the expression between the circRNAs and their parent genes in plants seems complicated, which merits further study. Despite that some circRNAs play roles by acting as miRNA sponges, very few of the identified circRNAs contain miRNA binding sites^12, 22, 31^. Herein by searching miRNA homologs within the database miRBase (http://www.mirbase.org/search.shtml), we found no miRNA targeting PSY1-circ1 (within miRNAs in *Solanum lycopersicum*, *Solanum tuberosum*, and *Nicotiana tabacum*). Therefore, there might be little functional potential of PSY1-circ1 action by miRNA inhibition.

As mentioned in another report^28^, we overexpressed circRNAs by introducing endogenous reverse complementary intronic sequences. The overexpression vector could generate the exact circRNAs with high abundance, however, it inevitably produced linear RNAs as well, although with much lower abundance. The linear RNA byproducts may interfere the function analysis of circRNAs. We found that both the circRNAs and the linear isoforms showed correlation with their parent mRNAs. It seems that the continuous high expression of PSY-circ1 or PDS-circ1 is related to their parent gene downregulation. However, the linear RNAs generated from the overexpression vectors may also contribute to the parent gene inhibition by co-suppression^46^.

In summary, we identified a large number of putative circRNAs, and characterized a few circRNAs derived from genes involved in the fruit pigment accumulation. *PSY1* derived circRNA and/or its linear isoforms could negatively regulate the expression of their parent gene, and reduce lycopene and β-carotene accumulation in tomato fruits. These discoveries reveal the potential circRNA behavior in fruit ripening, and provide evidence for the biological implication of circRNAs in plants. Future work is needed to reveal the mechanism of down-regulation of parent mRNAs in the transgenic lines over-expression plant circRNAs. In addition, since circRNAs could be inhibited by siRNA approaches^13^, knockdown of PSY1-circ1 by introducing artificial miRNA (amiRNA) targeting the back-splice site might provide further evidence of the functional role of PSY1-circ1 during fruit ripening.

## Methods

### Plant materials and growth conditions

Tomato (*Solanum lycopersicum* L.) plants (cvs. Ailsa Craig and microTom) were grown under growth room conditions (24 °C, 75% relative humidity, 16/8-h light/dark cycle, 500 μmol m^−2^ s^−1^ photosynthetic photon flux density). The seeds were surface sterilized with 5% sodium hypochlorite (w/v) for 25 minutes, washed with sterilized water for six times, and then sown in pots containing nutritive soil.

### Deep sequence of circular RNAs

Tomato (cv. Ailsa Craig) fruit pericarps at mature green, breaker, and breaker +6 stages were sampled for circular RNA detection (two independent biological replicates). Total RNA was extracted using Trizol reagent (Life Technologies, Carlsbad, CA). The rRNA was removed by a Ribo-Zero Magnetic kit (Epicentre, Madison, WI), and linear RNA was digested by RNase R (Epicentre, Madison, WI) at 37 °C for one hour. RNA-Seq library was prepared using TruSeq RNA LT Sample Prep kit v2 (Illumina, San Diego, CA) with insert length of 120–250 bp, and high-throughput sequencing was performed on HiSeq 2500 instrument with 100 bp paired-end reads (Genenergy Biotechnology Co., Ltd, Shanghai, China). The adaptors and low quality data were removed from the sequencing raw data using Trimmomatic (version 0.32) (parameters: ILLUMINACLIP: adaptor.fa:2:30:10 LEADING:3 TRAILING:3 SLIDINGWINDOW:4:15 MINLEN:50). The software Segemehl (v 0.1.7) and CIRI (v 2.0.5) were used for potential back-splice sites extraction^41, 42^.

### RNase R resistance test

Total RNA was isolated by Trizol reagent (Life Technologies, Carlsbad, CA) followed by DNA residue depletion using DNase I (Fermentas, Glen Burnie, MD). After purification by RNA Clean & Concentrator kit (Zymo Research, Irvine, CA), the RNA samples were separated to two aliquots, one aliquot was digested with RNase R (Epicentre, Madison, WI) at 37 °C for one hour (R+), the other aliquot was mock-treated with water (R−). Both R+ and R− were reverse transcribed (M-MLV Reverse Transcriptase, Promega, Madison, WI) to cDNA using random primers and used as templates for circRNA identification. Convergent and divergent primers (Supplementary Table 5) were used for linear and circRNA amplification respectively. Genomic DNA was also used as template to make certain that the site is not existent in genome.

### Quantitative real-time PCR analysis

Total RNA of tomato fruit pericarp was isolated using Trizol reagent followed by DNase I digestion at 37 °C for 30 minutes, then reverse transcribed to cDNA using random primers and used as templates for Real-time PCR. At least one of the divergent primers spanned the back-splice site, and the 3′ end of the primer that cross the junction was 3–6 bp in length. Real-time PCR was performed using the SYBR Select Master Mix on QuantStudio 6 Flex Real-time PCR System (Applied Biosystems, Foster City, CA). The primers used were listed in Supplementary Table 5.

### Vector construction and genetic transformation

To construct a vector for PSY1-circ1 overexpression, a genomic fragment consisting of the exons to be cyclized and its flanking sequences (281 bp upstream and 681 downstream) was cloned, then part of the downstream sequence (517 bp) was amplified. The two fragments were introduced into the expression vector pCAMBIA1301 in the opposite orientation under CaMV 35 S promoter (Supplementary Fig. 4). The PDS-circ1 overexpression vector was constructed in the same way as PSY1-circ1, the upstream and downstream flanking sequences of the circularized exons are 465 bp and 660 bp respectively, and the reverse inserted fragment from the downstream flanking intron is 462 bp. The vectors were introduced into *A. tumefaciens* EHA105, which was subsequently used to transform tomato Ailsa Craig and microTom following punctured cotyledon transformation method^47^.

### Lycopene and β-carotene content analysis

The fruit pericarp samples were excised, frozen with liquid nitrogen and stored at −80 °C. The frozen samples were ground to fine powder with liquid nitrogen, and approximately 0.2 g sample were extracted with 1.8 ml acetone-hexane (2:3) solvent for one hour. The optical density of the crude extract at 663 nm, 645 nm, 505 nm, and 453 nm were measured at the same time, using a UV-1800 spectrophotometer (Shimadzu, Kyoto, Japan). The contents of lycopene and β-carotene were estimated using the follow equations^48^: Lycopene (100 mg/100 mL) = −0.0458 A_663_ + 0.204 A_645_ + 0.372 A_505_ − 0.0806 A_453_, β-carotene (100 mg/100 mL) = 0.216 A_663_ − 1.22 A_645_ − 0.304 A_505_ + 0.452 A_453_.

## Electronic supplementary material

Supplementary Information
