# Supplementary material for: Identification and Functional Characterization of Tomato CircRNAs Derived from Genes Involved in Fruit Pigment Accumulation
Source: Sci Rep. 2017 Aug 17;7:8594. doi: 10.1038/s41598-017-08806-0 (PMC5561264; doi:10.1038/s41598-017-08806-0)
Supplement: Supplementary file 1 — Supplementary Information [file 41598_2017_8806_MOESM1_ESM.pdf]

Supplementary Material

Identification and Functional Characterization of Tomato CircRNAs Derived from Genes Involved in Fruit Pigment Accumulation

Jinjuan Tan[1], Zhongjing Zhou[1], Yujie Niu[1], Xiaoyong Sun[2], Zhiping Deng[1*]
[1] State Key Laboratory Breeding Base for Zhejiang Sustainable Pest and Disease Control, Institute of Virology and Biotechnology, Zhejiang Academy of Agricultural Sciences, Hangzhou 310021, China
[2] Agricultural Big-Data Research Center, College of Information Science And Engineering, Shandong Agricultural University, Taian, Shandong 271018, China
[*]email: zhipingdeng@126.com

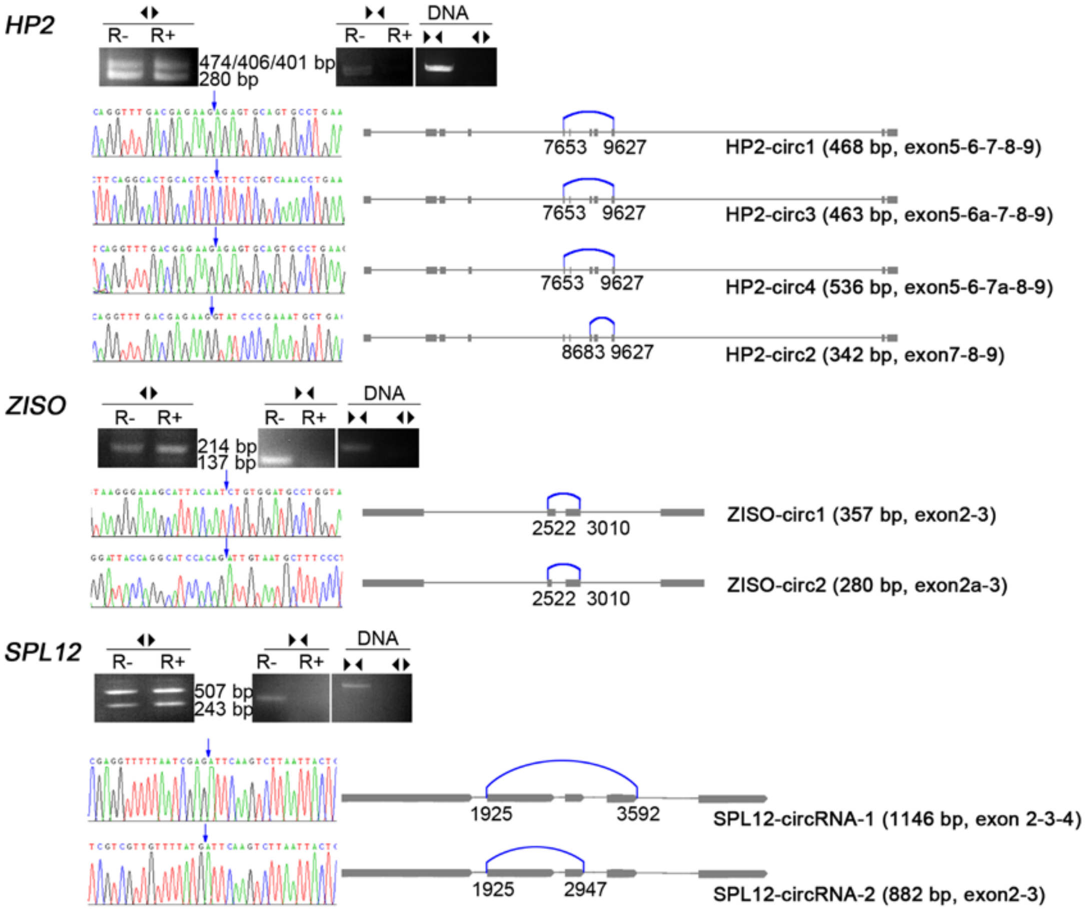

Supplementary Figure 1 Alternative circularization of circRNAs in tomato fruits. Alternative back-splicing circularization referring to different back-splice sites generated from the same gene was molecularly validated and showed in circRNAs derived from *HP2* and *SPL12* genes. Alternative splicing circularization referring to different transcripts with the same back-splice site was molecularly validated and showed in circRNAs derived from *HP2* and *ZISO* genes.

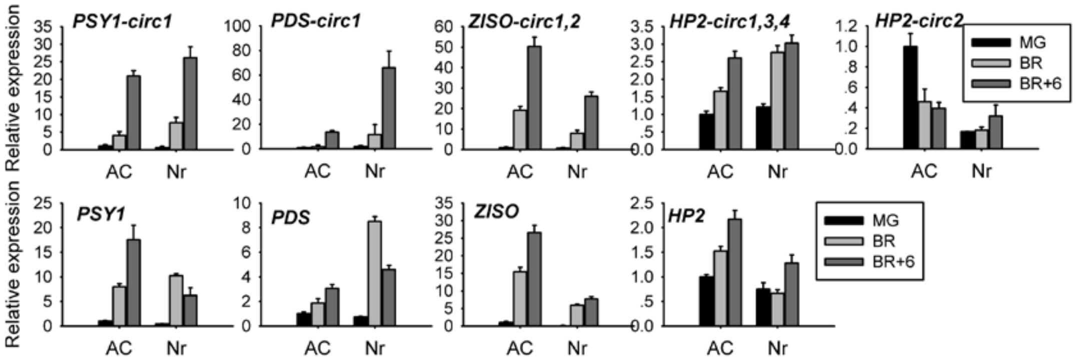

Supplementary Figure 2 Expression profile of a few circRNAs and their parent genes in Ailsa Craig and *Nr* mutant fruits at different ripening stages. The data were normalized to *Actin* and presented as the means ± SD (n=3).

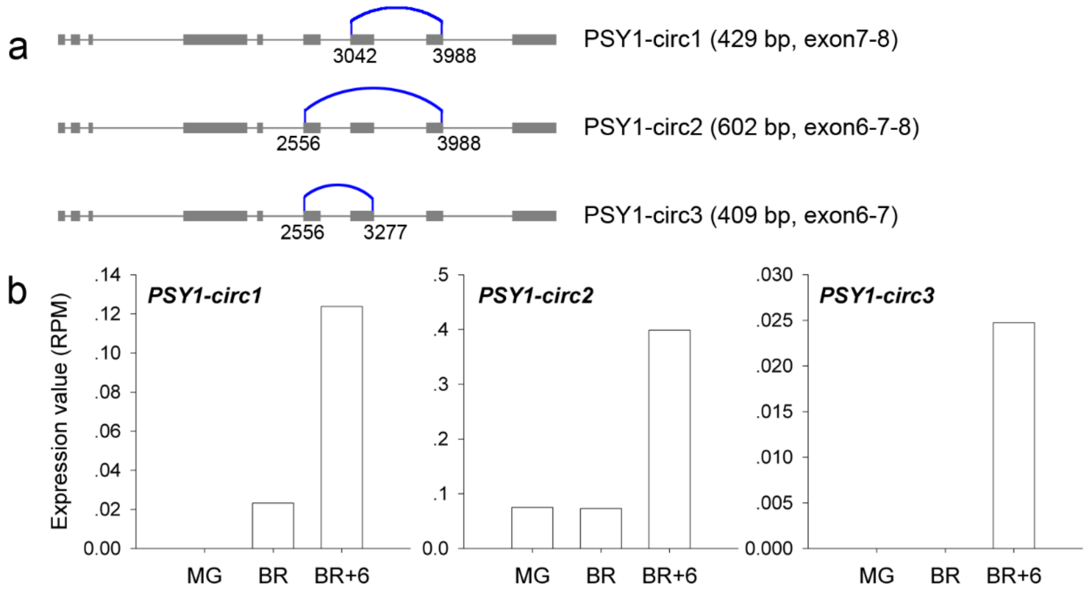

Supplementary Figure 3 Structure and expression profile of canonical circRNAs derived from *PSY1* calculated from RNA-seq data. Blue curves indicate canonical back-splice sites. RPM, reads per million mapped reads.

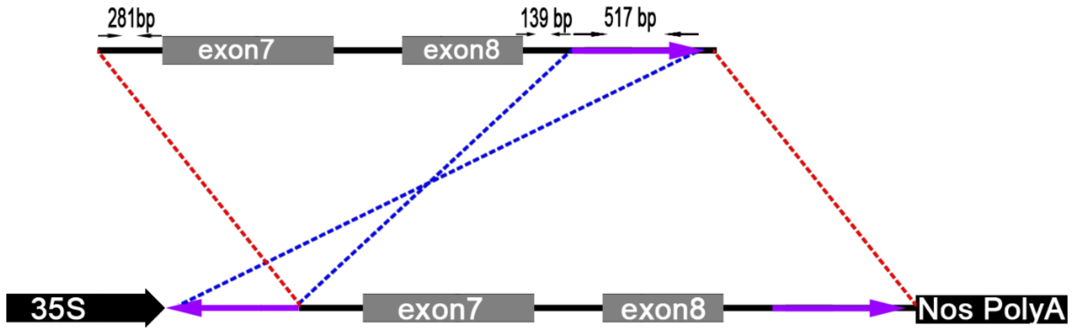

Supplementary Figure 4 Schematic diagram of vector construction for PSY1-circ1 overexpression. An intronic fragment (purple arrow) from the downstream flanking intron of the circularized PSY1-circ1 exons were introduced to the upstream flanking sequence in an opposite orientation, then the tandem fragments were introduced to pCAMBIA1301 vector to replace the *β-Glucuronidase* (*GUS*) gene, which is under drive of the CaMV 35S promoter.

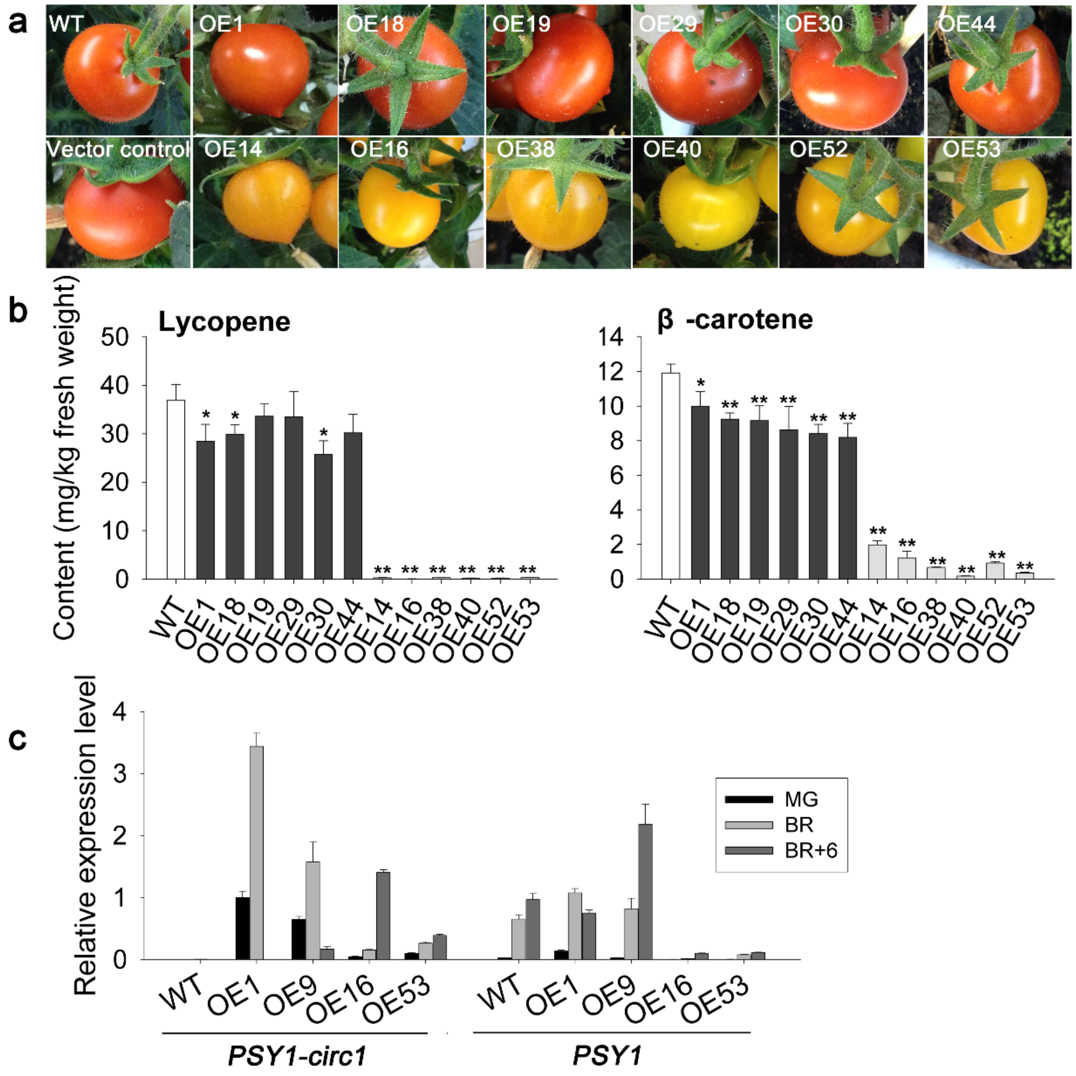

Supplementary Figure 5 Overexpression of PSY1-circ1 in microTom. (a) Fruits of different PSY1-circ1 overexpression tomato lines. WT, wild type. OE1-OE53 stand for transgenic lines. Vector control stands for microTom plants transformed with the empty vector pCAMBIA1301. (b) Lycopene and β-carotene contents in transgenic tomato fruits at breaker+10 stage. White, black and gray bars stand for control, transgenic lines with red fruits and yellow fruits respectively. The data with error bars were expressed as mean±SEM (n=4), asterisks indicate statistically significant difference (with respect to control; **p* value < 0.05; ***p* value <0.01; two-way ANOVA followed by LSD test). (c) Expression profile of PSY1-circ1 and *PSY1* gene in transgenic tomato fruits at different ripening stages. The data were normalized to *Actin* and presented as means ± SD (n=5).

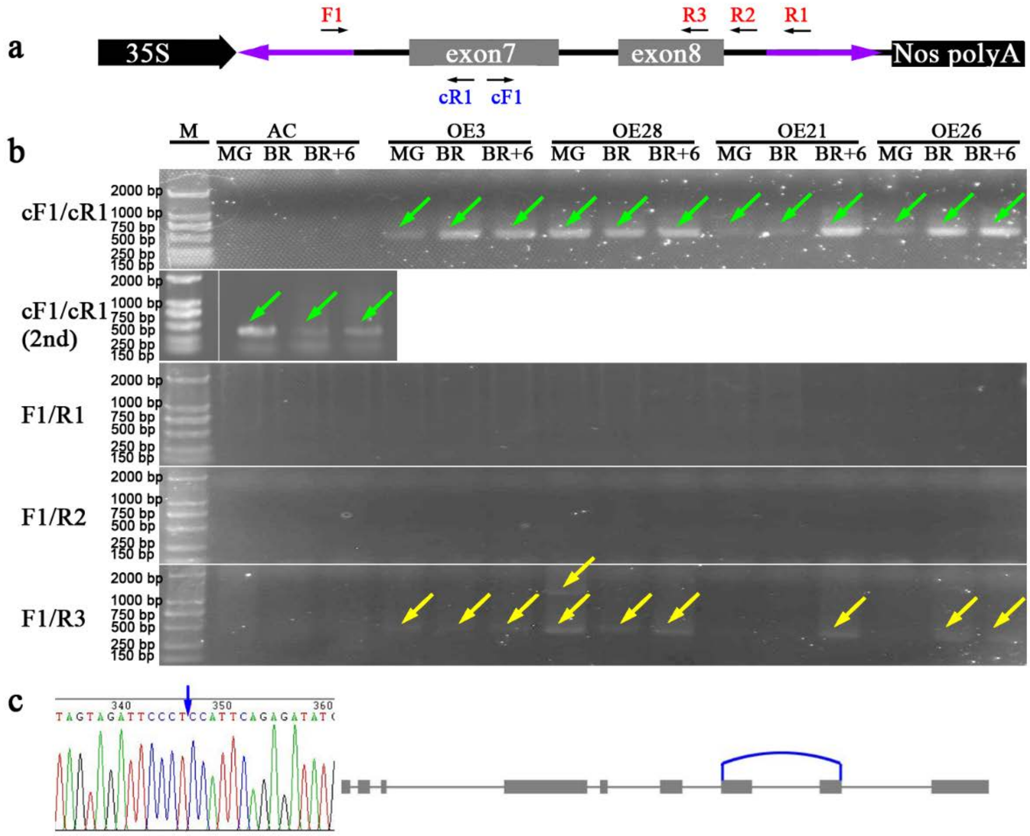

Supplementary Figure 6 Identification of RNA isoforms generated by PSY1-circ1 overexpressing vector. (a) Convergent primers F1/R1, F1/R2, and F1/R3 were used for amplification of linear RNAs, and divergent primers cF1/cR1 were used for circular RNA PSY1-circ1 identification. (b) PSY1-circ1 RNAs were successfully amplified in all fruit samples (green arrows), although in Ailsa Craig fruits the PCR products were only visible in the second-round PCR (used ten times diluted 1st PCR products as templates), since the first-round products were too low. Linear RNAs were also detected in some transgenic fruits (yellow arrows), but with lower abundance compared to PSY1-circ1. All PCRs were amplified with 35 cycles, and an equal aliquot of cDNA templates was used among PCRs with different primers. (c) Sanger sequencing of PCR products from (b, cF1/cR1) confirmed the back-splice site and structure of PSY1-circ1. The blue arrow and blue curve indicate the back-splice site.

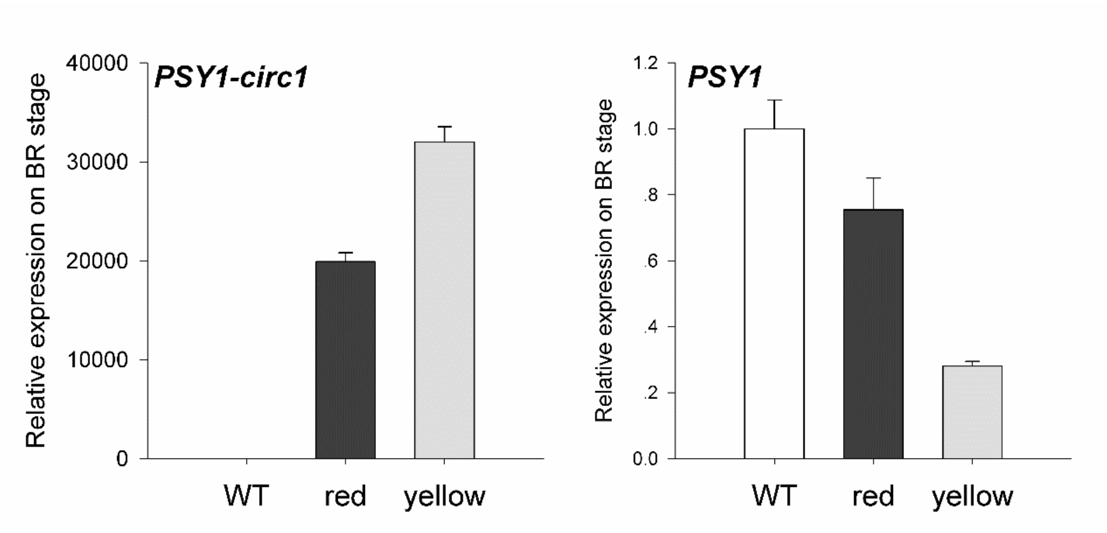

Supplementary Figure 7 Expression of PSY1-circ1 and its parent gene *PSY1* in transgenic microTom fruits (T1 generation). White, black and gray bars represent microTom control (WT), transgenic plants (T1 generation from OE1) with red fruits and yellow fruits. The data were normalized to *Actin* and are presented as the means ± SD (n=3).

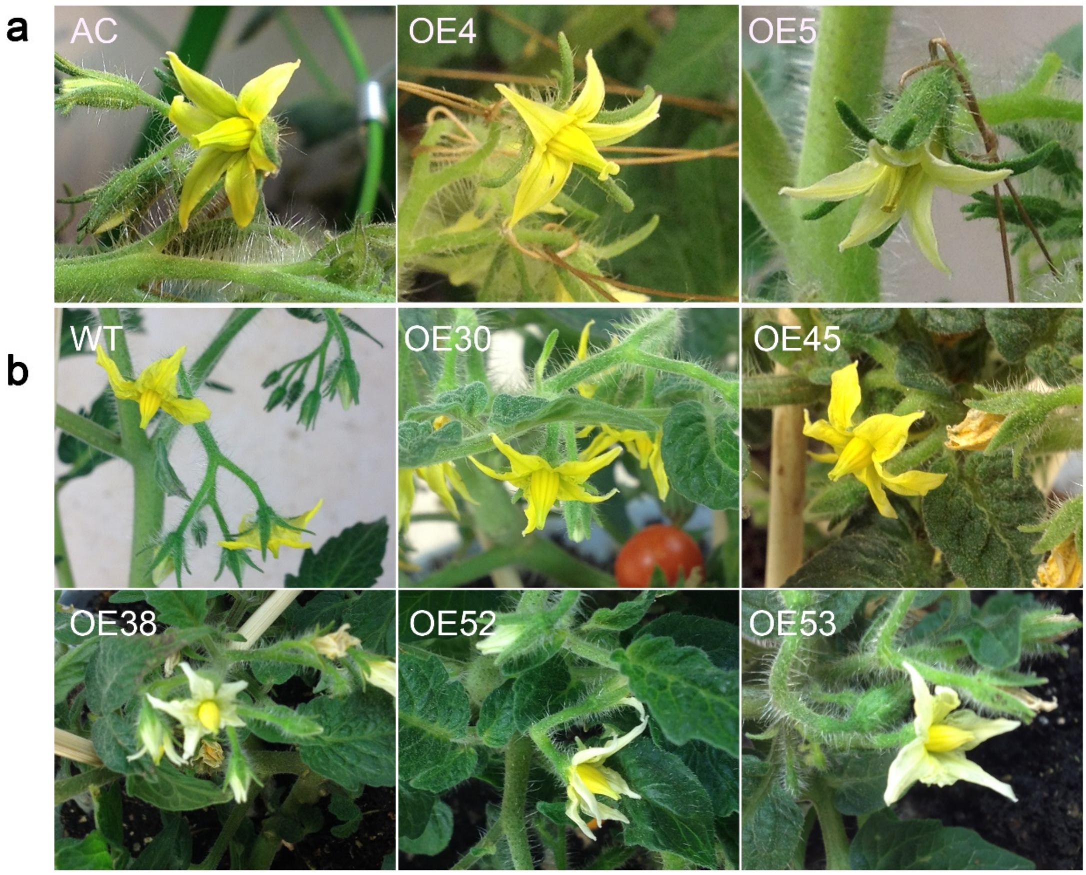

Supplementary Figure 8 Flowers of some PSY1-circ1 transgenic plants (Ailsa Craig and microTom). (a) OE4 is a transgenic Ailsa Craig line with yellow petals and red fruits, while OE5 is a transgenic line with white petals and yellow fruits. (b) OE30 and OE45 are transgenic microTom lines with yellow petals and red fruits, while OE38, OE52, and OE53 are transgenic lines with white petals and yellow fruits.

Supplementary Table 1 Putative circular RNAs identified in this study. The putative circRNAs identified by both Segemehl and CIRI were listed.

Chr	start of junction	end of junction
SL2.50ch12	64101116	64102065
SL2.50ch07	238342	238913
SL2.50ch01	958670	964383
SL2.50ch01	89425896	89429084
SL2.50ch09	1601732	1604230
SL2.50ch08	55342739	55343194
SL2.50ch01	89006909	89008059
SL2.50ch01	95428043	95428362
SL2.50ch02	44391539	44392572
SL2.50ch07	230400	231427
SL2.50ch02	54932573	54933227
SL2.50ch03	48957675	48958615
SL2.50ch07	210427	211962
SL2.50ch07	55511490	55512517
SL2.50ch08	14488119	14489541
SL2.50ch08	27200728	27203344
SL2.50ch08	55522642	55526314
SL2.50ch01	95753450	95753772
SL2.50ch07	238342	238616
SL2.50ch04	2475874	2478587
SL2.50ch04	63131174	63131614
SL2.50ch07	57180401	57180666
SL2.50ch07	57180401	57181056
SL2.50ch03	47811257	47813406
SL2.50ch04	63077366	63077665
SL2.50ch07	238342	239507
SL2.50ch12	4022638	4026890
SL2.50ch07	10358252	10376859
SL2.50ch09	1151845	1152244
SL2.50ch01	1435097	1436298
SL2.50ch01	6462808	6464408
SL2.50ch01	56450581	56451256
SL2.50ch01	80128239	80128990
SL2.50ch01	96218593	96219193
SL2.50ch04	25435531	25438441
SL2.50ch05	29311450	29314416
SL2.50ch06	3159038	3162413
SL2.50ch12	12005572	12006249
SL2.50ch05	45934021	45944368
SL2.50ch05	45944305	45950722
SL2.50ch06	35676533	35676817
SL2.50ch06	39016249	39016761
SL2.50ch07	238342	244716
SL2.50ch07	32781216	32791781
SL2.50ch08	63801024	63803144
SL2.50ch10	58199700	58200682
SL2.50ch11	39231669	39232094
SL2.50ch12	24504743	24508130
SL2.50ch12	66717368	66718592
SL2.50ch00	8482082	8484045
SL2.50ch04	3422619	3423369
SL2.50ch05	45934021	45950722
SL2.50ch06	3511784	3512526
SL2.50ch08	1472887	1474735

SL2.50ch09	64524037	64526784
SL2.50ch09	67723782	67724089
SL2.50ch01	1337381	1337968
SL2.50ch01	69463823	69473553
SL2.50ch02	54045625	54048832
SL2.50ch03	3785197	3786267
SL2.50ch03	4459901	4460783
SL2.50ch04	6174779	6176961
SL2.50ch04	20803914	20805074
SL2.50ch05	8869560	8872789
SL2.50ch07	230400	238616
SL2.50ch07	54091673	54092321
SL2.50ch08	54134174	54135136
SL2.50ch08	58617055	58617645
SL2.50ch09	12713068	12714300
SL2.50ch09	67463751	67464060
SL2.50ch11	6613040	6614625
SL2.50ch11	31576429	31583036
SL2.50ch01	39929846	39935529
SL2.50ch01	77199310	77201063
SL2.50ch01	79530151	79531226
SL2.50ch01	80715471	80721705
SL2.50ch01	83862289	83862924
SL2.50ch01	88329901	88332178
SL2.50ch01	89425896	89428528
SL2.50ch01	96218593	96219038
SL2.50ch01	98285985	98294960
SL2.50ch02	52531706	52536149
SL2.50ch03	4656225	4656587
SL2.50ch03	70521861	70522418
SL2.50ch04	50312838	50316571
SL2.50ch04	58278505	58279929
SL2.50ch05	1387906	1388664
SL2.50ch05	22630066	22635161
SL2.50ch05	22692128	22693249
SL2.50ch05	64816452	64816923
SL2.50ch06	63719	67191
SL2.50ch07	2327176	2327639
SL2.50ch07	39311827	39323602
SL2.50ch07	65990926	65993353
SL2.50ch08	65250628	65251280
SL2.50ch09	4050455	4050853
SL2.50ch09	33631019	33632615
SL2.50ch09	69703827	69704828
SL2.50ch10	26423257	26427890
SL2.50ch11	55935173	55942126
SL2.50ch00	549227	551528
SL2.50ch01	56464466	56478152
SL2.50ch01	75867821	75868153
SL2.50ch01	78830026	78831262
SL2.50ch01	79409311	79410342
SL2.50ch01	80628702	80629523
SL2.50ch01	85239105	85240959
SL2.50ch01	85763905	85766098
SL2.50ch01	86512179	86512804
SL2.50ch01	91286806	91287967
SL2.50ch02	40464307	40466985
SL2.50ch02	49700564	49701130

SL2.50ch03	56243027	56243877
SL2.50ch03	62201975	62202744
SL2.50ch03	63106220	63106929
SL2.50ch03	63444854	63451714
SL2.50ch03	69674991	69675715
SL2.50ch04	35325940	35337488
SL2.50ch04	62343892	62346454
SL2.50ch04	64386724	64387436
SL2.50ch05	2622349	2623431
SL2.50ch05	4080912	4083836
SL2.50ch05	9778479	9779633
SL2.50ch05	65836892	65863220
SL2.50ch05	65858043	65863655
SL2.50ch06	84413	86695
SL2.50ch06	39016249	39016565
SL2.50ch07	482888	484009
SL2.50ch07	21578418	21580627
SL2.50ch07	21578418	21593440
SL2.50ch07	39311827	39327145
SL2.50ch07	58598297	58600132
SL2.50ch07	62096067	62096482
SL2.50ch08	58130642	58131977
SL2.50ch08	62854165	62857677
SL2.50ch09	5105261	5106104
SL2.50ch09	68472198	68472917
SL2.50ch10	1445232	1446862
SL2.50ch10	10719501	10720338
SL2.50ch10	62750898	62752162
SL2.50ch10	64409259	64410267
SL2.50ch11	691678	694129
SL2.50ch11	9789910	9793557
SL2.50ch11	38098982	38105471
SL2.50ch11	52959123	52960176
SL2.50ch12	40792202	40792817
SL2.50ch12	53708885	53712558
SL2.50ch12	64399635	64403790
SL2.50ch12	66108134	66111069
SL2.50ch00	10340126	10342622
SL2.50ch01	1370040	1371007
SL2.50ch01	36258097	36261776
SL2.50ch01	56472387	56478152
SL2.50ch01	69624047	69628722
SL2.50ch01	75612495	75613798
SL2.50ch01	77848007	77849758
SL2.50ch01	81659714	81661855
SL2.50ch01	83846390	83848908
SL2.50ch01	86556968	86558817
SL2.50ch01	90838795	90840911
SL2.50ch01	97959539	97960266
SL2.50ch01	98168037	98168188
SL2.50ch02	4518110	4521555
SL2.50ch02	20243404	20246643
SL2.50ch02	35843192	35844046
SL2.50ch02	38655174	38657518
SL2.50ch02	48019848	48021515
SL2.50ch02	48021598	48021792
SL2.50ch02	51668642	51669278
SL2.50ch02	54045625	54047238

SL2.50ch03	4327521	4328953
SL2.50ch03	4659439	4661614
SL2.50ch03	11446124	11447307
SL2.50ch03	13204581	13207936
SL2.50ch03	35186044	35187019
SL2.50ch03	59167210	59168413
SL2.50ch03	66032747	66034299
SL2.50ch04	2372079	2376972
SL2.50ch04	7132219	7132546
SL2.50ch04	7583297	7584866
SL2.50ch04	41784452	41798922
SL2.50ch04	50864864	50865328
SL2.50ch04	58076013	58076614
SL2.50ch04	60063512	60066327
SL2.50ch04	64318664	64320196
SL2.50ch04	64387040	64387436
SL2.50ch05	3974975	3977801
SL2.50ch05	11607742	11608482
SL2.50ch05	20092602	20093576
SL2.50ch05	22621180	22625501
SL2.50ch05	22688256	22693249
SL2.50ch06	3160960	3162413
SL2.50ch06	19813189	19814983
SL2.50ch06	25811982	25812584
SL2.50ch06	32688949	32690149
SL2.50ch07	1028873	1031229
SL2.50ch08	2067344	2068198
SL2.50ch08	38134953	38143425
SL2.50ch08	52597389	52598271
SL2.50ch08	59525965	59529636
SL2.50ch09	1511373	1513107
SL2.50ch09	5180204	5180590
SL2.50ch09	16657456	16659160
SL2.50ch09	44421911	44428732
SL2.50ch09	44425933	44428732
SL2.50ch09	50118267	50120093
SL2.50ch09	50118428	50120093
SL2.50ch09	52232372	52240992
SL2.50ch10	92386	93435
SL2.50ch10	26423257	26425950
SL2.50ch11	3111530	3112158
SL2.50ch11	3486437	3487470
SL2.50ch11	6210415	6211030
SL2.50ch11	7250129	7250974
SL2.50ch11	7975429	7977329
SL2.50ch11	26363404	26364363
SL2.50ch11	31559100	31561356
SL2.50ch11	33338511	33342055
SL2.50ch11	38103925	38105471
SL2.50ch11	51716320	51717041
SL2.50ch11	52588849	52589446
SL2.50ch11	54550673	54551537
SL2.50ch11	55761855	55764385
SL2.50ch12	1829352	1830657
SL2.50ch12	3356416	3356789
SL2.50ch12	4689775	4692120
SL2.50ch12	43042562	43045200
SL2.50ch07	66924324	66925383

SL2.50ch10	393403	394744
SL2.50ch04	3422764	3423369
SL2.50ch05	16830716	16831649
SL2.50ch05	7109423	7110418
SL2.50ch07	4367529	4369821
SL2.50ch06	34378624	34379233
SL2.50ch02	4089484	4091023
SL2.50ch12	4658515	4661158
SL2.50ch01	55928143	55929350
SL2.50ch03	2720567	2724338
SL2.50ch05	19087698	19091131
SL2.50ch06	43764289	43764808
SL2.50ch06	44240777	44241611
SL2.50ch07	10520963	10524742
SL2.50ch09	67723517	67724089
SL2.50ch10	58308755	58314713
SL2.50ch10	59562047	59563404
SL2.50ch12	5164094	5167332
SL2.50ch01	88125455	88128447
SL2.50ch01	93367456	93368439
SL2.50ch02	47702068	47704681
SL2.50ch03	48957675	48958181
SL2.50ch04	16923096	16923456
SL2.50ch04	54569894	54576288
SL2.50ch05	29298869	29314416
SL2.50ch06	37608020	37608275
SL2.50ch07	63777493	63779564
SL2.50ch07	67890741	67891151
SL2.50ch08	25453816	25455622
SL2.50ch08	58719803	58720231
SL2.50ch10	14097625	14098018
SL2.50ch10	21871504	21872211
SL2.50ch10	26423257	26425893
SL2.50ch10	63374224	63374810
SL2.50ch11	4489225	4493646
SL2.50ch11	37785891	37787454
SL2.50ch11	49241833	49243434
SL2.50ch11	52164260	52165291
SL2.50ch12	62524504	62525340
SL2.50ch01	950146	952323
SL2.50ch01	2881585	2882446
SL2.50ch01	41410267	41411251
SL2.50ch01	91734869	91736920
SL2.50ch01	91899495	91901886
SL2.50ch01	93195574	93198269
SL2.50ch01	96337246	96340051
SL2.50ch01	97801204	97801452
SL2.50ch02	1275452	1276462
SL2.50ch02	25128030	25128299
SL2.50ch02	46351393	46352213
SL2.50ch02	48938202	48939789
SL2.50ch02	55233897	55235434
SL2.50ch03	1681002	1687262
SL2.50ch03	32481569	32501726
SL2.50ch03	50080745	50081194
SL2.50ch04	7376093	7380752
SL2.50ch04	33536561	33538568
SL2.50ch04	59880627	59884881

SL2.50ch04	61025224	61027063
SL2.50ch04	63968504	63970772
SL2.50ch05	45948902	45950722
SL2.50ch05	54978089	54981054
SL2.50ch05	65858043	65859273
SL2.50ch06	3285075	3286464
SL2.50ch06	32941228	32941746
SL2.50ch06	44241308	44242443
SL2.50ch07	6942081	6943038
SL2.50ch07	10521238	10524742
SL2.50ch07	21601223	21602675
SL2.50ch07	53435253	53435854
SL2.50ch07	58397818	58398716
SL2.50ch07	59497949	59502029
SL2.50ch08	14488122	14489541
SL2.50ch08	54124803	54125996
SL2.50ch08	54124803	54135136
SL2.50ch08	58407097	58408235
SL2.50ch08	59469259	59471246
SL2.50ch09	1594407	1595844
SL2.50ch10	2080706	2082578
SL2.50ch10	26423257	26427741
SL2.50ch10	62365104	62366908
SL2.50ch11	39243500	39245882
SL2.50ch11	41915754	41916262
SL2.50ch11	43309417	43310303
SL2.50ch11	48754790	48758917
SL2.50ch12	1611519	1614586
SL2.50ch12	53672516	53673269
SL2.50ch12	53700671	53711562
SL2.50ch12	65121620	65127107
SL2.50ch01	70014	70628
SL2.50ch01	93625	96370
SL2.50ch01	72321459	72325995
SL2.50ch01	76660710	76662457
SL2.50ch01	96218067	96218521
SL2.50ch01	96218593	96218838
SL2.50ch02	34504694	34507171
SL2.50ch02	37246980	37248816
SL2.50ch02	44414628	44417028
SL2.50ch02	46421709	46424050
SL2.50ch02	47819578	47820961
SL2.50ch02	48328075	48328735
SL2.50ch02	51851151	51851623
SL2.50ch02	53774333	53774630
SL2.50ch02	54242184	54242960
SL2.50ch02	54948678	54949496
SL2.50ch03	1366330	1367896
SL2.50ch03	1367588	1367896
SL2.50ch03	4656225	4657119
SL2.50ch03	18205552	18206254
SL2.50ch03	32497948	32501726
SL2.50ch03	48954691	48958615
SL2.50ch03	65492033	65492448
SL2.50ch04	1405958	1408197
SL2.50ch04	58075423	58076220
SL2.50ch04	58278505	58282665
SL2.50ch04	61423965	61424408

SL2.50ch05	1609830	1610838
SL2.50ch05	2422026	2422361
SL2.50ch05	3800601	3801014
SL2.50ch05	5784202	5786570
SL2.50ch05	9732687	9734098
SL2.50ch05	29304007	29314416
SL2.50ch05	59108375	59109056
SL2.50ch05	65850081	65863655
SL2.50ch06	35873857	35874538
SL2.50ch06	41972841	41973223
SL2.50ch06	41993192	41994001
SL2.50ch06	43636754	43639154
SL2.50ch06	46443513	46444703
SL2.50ch07	10526081	10527102
SL2.50ch07	21549013	21561488
SL2.50ch07	63690661	63691261
SL2.50ch07	63847262	63849385
SL2.50ch08	51793930	51803201
SL2.50ch08	55522642	55523200
SL2.50ch08	56908325	56914404
SL2.50ch08	59469259	59469864
SL2.50ch08	59888334	59891038
SL2.50ch08	60088604	60089839
SL2.50ch08	60089356	60093223
SL2.50ch08	62133379	62134382
SL2.50ch08	65167593	65172584
SL2.50ch09	802422	804823
SL2.50ch09	1654384	1654610
SL2.50ch09	3375762	3376180
SL2.50ch09	4662997	4663797
SL2.50ch09	16657784	16659160
SL2.50ch09	42263225	42263978
SL2.50ch09	65661120	65662030
SL2.50ch09	68496300	68497532
SL2.50ch09	71034252	71035810
SL2.50ch10	58292365	58292868
SL2.50ch11	4210343	4210799
SL2.50ch11	31563054	31573242
SL2.50ch11	32943437	32946305
SL2.50ch11	50811334	50812180
SL2.50ch12	217442	217915
SL2.50ch12	2065962	2066920
SL2.50ch12	3566900	3567533
SL2.50ch12	10409242	10410399
SL2.50ch12	64401220	64403790
SL2.50ch12	64996487	65000697
SL2.50ch12	65188991	65189273
SL2.50ch12	64171469	64172017
SL2.50ch09	1500972	1503561
SL2.50ch12	65475978	65476430
SL2.50ch08	1276168	1277561
SL2.50ch01	820898	822978
SL2.50ch01	86106056	86106704
SL2.50ch08	25694879	25696508
SL2.50ch08	54212037	54212838
SL2.50ch08	60997789	60999534
SL2.50ch11	5353386	5353797
SL2.50ch10	1307213	1307526

SL2.50ch01	53602896	53604870
SL2.50ch03	66167869	66168990
SL2.50ch06	84413	88510
SL2.50ch06	5405158	5406468
SL2.50ch09	69300335	69300656
SL2.50ch10	57048647	57051100
SL2.50ch11	119413	119977
SL2.50ch11	723254	728197
SL2.50ch11	43321339	43321664
SL2.50ch12	5159090	5159623
SL2.50ch12	40752081	40753680
SL2.50ch01	43301186	43301878
SL2.50ch01	82330208	82334863
SL2.50ch01	90630990	90635761
SL2.50ch02	38332994	38335216
SL2.50ch02	46148680	46149442
SL2.50ch02	54574060	54574355
SL2.50ch03	48957675	48958466
SL2.50ch03	52908503	52909510
SL2.50ch03	69998505	70000400
SL2.50ch05	472149	472586
SL2.50ch05	1098231	1100806
SL2.50ch05	60506583	60509059
SL2.50ch05	63150654	63150977
SL2.50ch06	38408116	38409846
SL2.50ch07	51886032	51886592
SL2.50ch07	51978020	51978765
SL2.50ch07	64404265	64405554
SL2.50ch08	23794785	23795898
SL2.50ch08	51704250	51705225
SL2.50ch08	61380487	61381154
SL2.50ch10	1064902	1065599
SL2.50ch11	6414685	6415189
SL2.50ch11	33341469	33342055
SL2.50ch11	55455170	55455539
SL2.50ch12	3819467	3820138
SL2.50ch12	15390991	15391501
SL2.50ch12	42547078	42548460
SL2.50ch12	66775482	66778458
SL2.50ch00	4305221	4305632
SL2.50ch01	1224426	1225750
SL2.50ch01	1291118	1291401
SL2.50ch01	48396128	48396446
SL2.50ch01	56475386	56478152
SL2.50ch01	81359434	81360413
SL2.50ch01	93676693	93678517
SL2.50ch01	95189874	95191854
SL2.50ch01	98292433	98294960
SL2.50ch01	98358813	98359417
SL2.50ch02	27234035	27234583
SL2.50ch02	29565234	29573580
SL2.50ch02	41368054	41369164
SL2.50ch02	43284367	43285814
SL2.50ch02	51188457	51190256
SL2.50ch03	61668443	61670417
SL2.50ch04	7583297	7584156
SL2.50ch04	59779836	59780749
SL2.50ch04	60233710	60234096

SL2.50ch04	61450428	61451963
SL2.50ch05	5042626	5043828
SL2.50ch05	23875277	23876744
SL2.50ch05	61184436	61184822
SL2.50ch05	63874443	63874933
SL2.50ch06	1760904	1761388
SL2.50ch06	37606914	37607937
SL2.50ch07	976732	977512
SL2.50ch07	7732585	7735844
SL2.50ch07	63869552	63872708
SL2.50ch08	2603215	2606808
SL2.50ch08	61692684	61693435
SL2.50ch08	65682545	65687123
SL2.50ch09	1273900	1274403
SL2.50ch09	1654611	1654980
SL2.50ch09	3824966	3825169
SL2.50ch10	792863	795612
SL2.50ch10	60588123	60588581
SL2.50ch11	5545234	5546917
SL2.50ch11	5964037	5965575
SL2.50ch11	6099480	6100805
SL2.50ch11	49276983	49277744
SL2.50ch11	50612451	50613955
SL2.50ch11	52222851	52223670
SL2.50ch11	55113648	55115281
SL2.50ch12	5163588	5167332
SL2.50ch12	12005572	12008556
SL2.50ch12	37883939	37888582
SL2.50ch12	62109878	62110173
SL2.50ch12	66507278	66512105
SL2.50ch07	59218379	59220089
SL2.50ch05	63418316	63419432
SL2.50ch01	76660275	76660892
SL2.50ch02	47800873	47805003
SL2.50ch08	60425246	60425698
SL2.50ch11	30393446	30396689
SL2.50ch01	90619882	90622529
SL2.50ch02	38096019	38097479
SL2.50ch04	16923096	16925145
SL2.50ch06	1805580	1806074
SL2.50ch06	43176125	43177400
SL2.50ch01	82777176	82780246
SL2.50ch01	89729655	89733533
SL2.50ch02	22716424	22718663
SL2.50ch03	64059405	64060002
SL2.50ch04	18259845	18272052
SL2.50ch05	10050308	10055285
SL2.50ch05	10063412	10066958
SL2.50ch05	16810911	16813250
SL2.50ch07	56496311	56496886
SL2.50ch08	55522642	55528313
SL2.50ch10	408375	409635
SL2.50ch10	10862536	10863685
SL2.50ch11	53734462	53744307
SL2.50ch12	37589810	37590042
SL2.50ch12	53519635	53520442
SL2.50ch12	66470736	66471292
SL2.50ch01	35350510	35354615

SL2.50ch01	83866611	83869250
SL2.50ch01	88759973	88764323
SL2.50ch01	90528374	90529066
SL2.50ch01	97103363	97105089
SL2.50ch02	1288194	1308248
SL2.50ch02	24613596	24616154
SL2.50ch02	50657274	50659209
SL2.50ch03	13176287	13179618
SL2.50ch03	59664353	59667292
SL2.50ch03	60523573	60527185
SL2.50ch03	62186809	62187576
SL2.50ch03	65355537	65357605
SL2.50ch04	627113	627743
SL2.50ch04	5362849	5363376
SL2.50ch04	5962291	5964041
SL2.50ch04	25067738	25068642
SL2.50ch04	33533730	33538568
SL2.50ch04	41770520	41772196
SL2.50ch04	55653007	55654852
SL2.50ch04	66230132	66232863
SL2.50ch05	19101465	19105008
SL2.50ch05	63350049	63350677
SL2.50ch06	2044518	2047758
SL2.50ch06	32768836	32770193
SL2.50ch06	43791629	43792446
SL2.50ch07	1268573	1269134
SL2.50ch07	2441518	2441759
SL2.50ch07	10375803	10376859
SL2.50ch07	64249019	64250668
SL2.50ch08	25491703	25492378
SL2.50ch08	55522642	55526071
SL2.50ch08	61288390	61289042
SL2.50ch09	2755542	2760130
SL2.50ch09	3556710	3569693
SL2.50ch09	64197764	64198629
SL2.50ch09	70260435	70265364
SL2.50ch10	92386	93935
SL2.50ch10	208225	208693
SL2.50ch10	58624104	58625733
SL2.50ch11	3356043	3356413
SL2.50ch11	7831727	7832592
SL2.50ch11	55193423	55194347
SL2.50ch12	3472364	3473572
SL2.50ch12	37739240	37739821
SL2.50ch01	80715471	80721707
SL2.50ch01	90605614	90607989
SL2.50ch09	67723512	67724089
SL2.50ch11	55524913	55525902
SL2.50ch11	53736751	53741379
SL2.50ch01	90630990	90633168
SL2.50ch09	72086110	72089608
SL2.50ch01	90947891	90950791
SL2.50ch04	55751401	55752727
SL2.50ch07	977078	977512
SL2.50ch07	10523474	10525759
SL2.50ch08	64142078	64143336
SL2.50ch09	1500972	1503099
SL2.50ch09	33689613	33691770

SL2.50ch11	41541444	41543579
SL2.50ch11	49247210	49248279
SL2.50ch01	1695810	1697212
SL2.50ch03	4328007	4328953
SL2.50ch03	61828193	61829642
SL2.50ch03	69945580	69948922
SL2.50ch07	59014613	59015192
SL2.50ch08	58401946	58405621
SL2.50ch08	58606436	58607082
SL2.50ch09	62488802	62490152
SL2.50ch10	11326837	11327338
SL2.50ch12	4533376	4533872
SL2.50ch01	13177	13661
SL2.50ch01	56472387	56475756
SL2.50ch01	80404610	80411443
SL2.50ch01	96699865	96700805
SL2.50ch02	25127863	25128299
SL2.50ch02	53580399	53581188
SL2.50ch03	52969572	52972893
SL2.50ch03	68944514	68945013
SL2.50ch04	62793474	62797977
SL2.50ch05	1387522	1388010
SL2.50ch05	6708895	6711557
SL2.50ch05	60344954	60349570
SL2.50ch06	40776441	40779461
SL2.50ch07	56332486	56335535
SL2.50ch08	6990847	6991816
SL2.50ch10	63788551	63789351
SL2.50ch11	36507684	36510824
SL2.50ch12	2352797	2354696
SL2.50ch12	66578393	66578819
SL2.50ch01	81898868	81899402
SL2.50ch01	84289805	84290540
SL2.50ch01	87477286	87482023
SL2.50ch01	92232690	92235858
SL2.50ch02	44770475	44772818
SL2.50ch02	54932573	54932950
SL2.50ch04	65620535	65625927
SL2.50ch05	46228872	46234509
SL2.50ch06	71344	73717
SL2.50ch06	31181398	31183175
SL2.50ch06	48983142	48984276
SL2.50ch07	4200218	4201053
SL2.50ch07	21389876	21391076
SL2.50ch08	1472887	1480923
SL2.50ch08	55522642	55525092
SL2.50ch09	2755542	2762584
SL2.50ch09	31893586	31896198
SL2.50ch09	31896266	31896797
SL2.50ch11	10033364	10034334
SL2.50ch12	1611519	1615179
SL2.50ch12	1620849	1624951
SL2.50ch12	29110650	29111090
SL2.50ch12	66108134	66110253
SL2.50ch01	1122379	1122719
SL2.50ch01	13979184	13979678
SL2.50ch01	54560020	54568676
SL2.50ch01	88125455	88126083

SL2.50ch02	34384699	34388946
SL2.50ch02	38287222	38287866
SL2.50ch02	42799160	42799339
SL2.50ch02	44817076	44817429
SL2.50ch02	46850433	46852137
SL2.50ch03	3009332	3010131
SL2.50ch03	8201277	8203496
SL2.50ch03	55183863	55197548
SL2.50ch03	62197202	62199662
SL2.50ch03	65242618	65243396
SL2.50ch03	66803073	66803827
SL2.50ch03	69770063	69773085
SL2.50ch03	70637392	70641648
SL2.50ch04	2264722	2270861
SL2.50ch04	18195180	18195633
SL2.50ch04	45321116	45322667
SL2.50ch04	60108702	60109162
SL2.50ch05	5225922	5227056
SL2.50ch05	65691748	65693428
SL2.50ch06	34134687	34135147
SL2.50ch06	42528895	42538395
SL2.50ch06	43174977	43177400
SL2.50ch06	43254780	43256242
SL2.50ch06	43638262	43641934
SL2.50ch06	48027396	48028196
SL2.50ch07	3730434	3737549
SL2.50ch07	6949732	6950824
SL2.50ch07	7668400	7669088
SL2.50ch07	21208786	21210064
SL2.50ch07	65842911	65843684
SL2.50ch08	54720018	54720661
SL2.50ch08	58407097	58409623
SL2.50ch09	5793547	5793920
SL2.50ch09	22837104	22837779
SL2.50ch09	31113070	31127995
SL2.50ch09	40063351	40066414
SL2.50ch09	48789677	48790242
SL2.50ch09	67151648	67155803
SL2.50ch10	890903	892387
SL2.50ch10	3051159	3051822
SL2.50ch10	18504927	18515676
SL2.50ch11	6614010	6614625
SL2.50ch11	25025604	25026201
SL2.50ch11	52805479	52805944
SL2.50ch12	61932127	61933038
SL2.50ch12	62055340	62055807
SL2.50ch12	64996487	65000252
SL2.50ch12	66004439	66005898
SL2.50ch12	66212221	66216669
SL2.50ch01	83846390	83847922
SL2.50ch01	90605614	90612102
SL2.50ch03	30990	32428
SL2.50ch08	46339680	46340152
SL2.50ch04	6532541	6534728
SL2.50ch12	66127788	66128276
SL2.50ch01	94484383	94487046
SL2.50ch02	49623047	49623805
SL2.50ch04	60065839	60066532

SL2.50ch07	68007493	68010577
SL2.50ch12	65128136	65129767
SL2.50ch01	89492725	89493458
SL2.50ch01	89730975	89733533
SL2.50ch06	36729690	36734553
SL2.50ch08	60432213	60433729
SL2.50ch09	69299833	69300656
SL2.50ch10	46300785	46302324
SL2.50ch01	673286	676121
SL2.50ch01	81306099	81308949
SL2.50ch02	43124517	43125172
SL2.50ch02	49502888	49503487
SL2.50ch03	68944597	68945013
SL2.50ch07	10523453	10527102
SL2.50ch08	58926211	58927066
SL2.50ch09	67135125	67136390
SL2.50ch12	10201277	10203029
SL2.50ch01	82651832	82654137
SL2.50ch01	87427188	87427482
SL2.50ch01	88165816	88166171
SL2.50ch02	23574028	23583508
SL2.50ch03	976173	979129
SL2.50ch03	24749210	24749624
SL2.50ch03	64453761	64456536
SL2.50ch03	70459987	70461341
SL2.50ch04	358448	358951
SL2.50ch04	65406403	65406865
SL2.50ch05	2577529	2579535
SL2.50ch05	58563460	58564809
SL2.50ch07	21578418	21593436
SL2.50ch07	67546087	67547159
SL2.50ch08	25829679	25831964
SL2.50ch08	64305831	64306523
SL2.50ch10	1889965	1891454
SL2.50ch10	14126858	14128072
SL2.50ch10	58507010	58507991
SL2.50ch11	49473872	49476376
SL2.50ch12	48120404	48122121
SL2.50ch01	75014690	75015790
SL2.50ch01	75465377	75468823
SL2.50ch01	81659771	81661855
SL2.50ch01	90949911	90950791
SL2.50ch01	93092828	93094156
SL2.50ch02	1284294	1289542
SL2.50ch02	51010954	51012186
SL2.50ch03	2927940	2932099
SL2.50ch03	4967203	4969713
SL2.50ch03	56799637	56801379
SL2.50ch04	2939441	2942441
SL2.50ch04	7376097	7380752
SL2.50ch04	29416785	29420401
SL2.50ch04	41784452	41785026
SL2.50ch06	76764	80638
SL2.50ch06	45900772	45902417
SL2.50ch07	61913265	61919613
SL2.50ch08	64146684	64148175
SL2.50ch10	19704196	19706313
SL2.50ch10	49988106	49993179

SL2.50ch10	58658191	58659219
SL2.50ch11	5534827	5535370
SL2.50ch11	7981451	7983985
SL2.50ch11	33971742	33972203
SL2.50ch11	52742483	52744792
SL2.50ch12	3437518	3437863
SL2.50ch12	26607299	26611856
SL2.50ch12	61691393	61691682
SL2.50ch01	3098317	3098878
SL2.50ch01	55928143	55931501
SL2.50ch01	72325238	72325995
SL2.50ch01	76871263	76871888
SL2.50ch01	89821301	89826056
SL2.50ch01	92249361	92253835
SL2.50ch01	92872760	92881110
SL2.50ch02	22862344	22864338
SL2.50ch02	52183957	52188681
SL2.50ch03	3511408	3516225
SL2.50ch03	53126735	53127804
SL2.50ch03	69952270	69953293
SL2.50ch04	209926	210329
SL2.50ch04	1208879	1214277
SL2.50ch05	534133	534597
SL2.50ch05	10082545	10089432
SL2.50ch06	15696210	15697299
SL2.50ch06	37606914	37607781
SL2.50ch06	40863584	40864081
SL2.50ch06	45008998	45009354
SL2.50ch06	46282005	46282353
SL2.50ch06	49661667	49666403
SL2.50ch07	279900	286050
SL2.50ch07	2482147	2491155
SL2.50ch07	7408758	7411930
SL2.50ch07	64111958	64112701
SL2.50ch08	1583168	1584778
SL2.50ch08	2379362	2381616
SL2.50ch10	468727	469595
SL2.50ch10	1081541	1086146
SL2.50ch10	2411566	2413051
SL2.50ch10	40326425	40327209
SL2.50ch10	58609556	58611315
SL2.50ch10	61947171	61948408
SL2.50ch11	8422662	8422939
SL2.50ch11	53734462	53741997
SL2.50ch12	29122638	29127812
SL2.50ch12	47338038	47338869

Supplementary Table 2 Molecularly identified circRNAs in Ailsa Craig fruits

Gene name	Circle length (bp)	Identical to bioinfromatic prediction	Splicing pattern	Cyclized exons*	circRNA name
ETR1	962	Yes	Canonical	exon 3-4-5-6	
	766	No	Non-canonical	exon 3a-4-5-6a	
ETR3	764	Yes	Canonical	exon 5-6-7	
	594	No	Non-canonical	exon 5a-6-7a	
	636	No	Canonical	exon 5-6	
ETR4	544	No	Non-canonical	exon 2a-3a	ETR4-circ1
	199	No	Non-canonical	exon 2b-3b	ETR4-circ2
EIL3	481	No	Non-canonical	exon 2a-2a	
	240	No	Non-canonical	exon 2b-2b	
	433	No	Non-canonical	exon 2c-2c	
JERF1	619	No	Non-canonical	exon 1a-2a	
	449	No	Non-canonical	exon 1b-2b	
	340	No	Non-canonical	exon 1c-2c	
	353	No	Non-canonical	exon 1d-2d	
ACO6	652	No	Non-canonical	exon 2a-3-4a	ACO6-circ1
	531	No	Non-canonical	exon 1a-2-3a	ACO6-circ2
	401	No	Non-canonical	exon 2b-3b	ACO6-circ3
	311	No	Non-canonical	exon 2c-3c	ACO6-circ4
CNR	90	No	Non-canonical	exon 3a-3a	
	69	No	Non-canonical	exon 3b-3b	
	281	No	Non-canonical	exon 2a-3c	
NAC3	314	No	Non-canonical	exon 2a-3a	
SPL1	644	No	Canonical	exon 2a-3	
	736	No	Canonical	exon 2-3	
	858	Yes	Canonical	exon 3-4-5-6	
SPL12	1146	Yes	Canonical	exon 2-3-4	SPL12-circ1
	882	No	Canonical	exon 2-3	SPL12-circ2
PSY1	316	No	Non-canonical	exon 8a-9a	
	227	No	Non-canonical	exon 8b-9b	
	270	No	Non-canonical	exon 7a-8-9c	
	316	No	Non-canonical	exon 8c-9d	
	429	Yes	Canonical	exon 7-8	PSY1-circ1
	405	No	Non-canonical	exon 6a-7-8d	
	510	No	Non-canonical	exon 5a-6-7-8e	
PDS	406	No	Non-canonical	exon 7a-8-9-10a	
	600	No	Non-canonical	exon 6a-7-8-9-10b	
	309	No	Non-canonical	exon 7b-8-9a	
	545	No	Non-canonical	exon 6b-7-8-9-10c	
	996	No	Non-canonical	exon 6-7-8-9-10-11-12-13-14a	
	645	No	Non-canonical	exon 6c-7-8-9-10d	
	571	No	Non-canonical	exon 6-7-8-9-10e	
	515	No	Non-canonical	exon 6d-7-8-9-10f	
	763	No	Non-canonical	exon 4a-5-6-7-8-9-10g	
	483	Yes	Canonical	exon 6-7-8-9	PDS-circ1
HP2	468	Yes	Canonical	exon 5-6-7-8-9	HP2-circ1
	342	No	Canonical	exon 7-8-9	HP2-circ2
	463	Yes	Canonical	exon 5-6a-7-8-9	HP2-circ3
	536	Yes	Canonical	exon 5-6-7a-8-9	HP2-circ4
ZISO	357	Yes	Canonical	exon 2-3	ZISO-circ1
	280	Yes	Canonical	exon 2a-3	ZISO-circ2

HP1	387	No	Non-canonical	exon 18a-18a
CCD1	249	No	Non-canonical	exon 12a-13-14a
	440	No	Non-canonical	exon 12b-13-14b

*: Lowercase a-g indicates alternative splicing sites

Supplementary Table 3 Circular RNAs derived from PSY1 gene

Samples	Type	Name	Back-splice site	POS1	POS2	INF	RPM
ACMG-1	canonical	PSY1-circ2	GGGGTAATATATGATGCGTTTGGGCCATCAACAAGTTCATCTGTTCTTCTGCACCATAAGGGAATCTACTAGCTGAGCTCAATTCTGTCACGCCTTTCTCT	4327521	4328953	splits:2:10:2:C:P	0.051
ACMG-2	canonical	PSY1-circ2	TGGGCCATCAACAAGTTCATCTGTTCTTCTGCACCATAAGGGAATCTACTAGCTGAGCTCAATTCTGTCACGCCTTTCTCTGCCTCATCAAAGAACTTTCT	4327521	4328953	splits:1:1:1:C:P	0.024
ACBR-1	non-canonical	PSY1-circ4	CGTACCAGCAACATAATAACAATAAAGGTATAGTTCGTCGAAGTTTTTAAGTTGTTGTAGTCATTGGCTTCAATCTCATCTAGTATTTTGCGGTACAAGAC	4328061	4329710	splits:2:2:2:C:P	0.046
	canonical	PSY1-circ2	AGAAAGGCGTGACAGAATTGAGCTCAGCTAGTAGATTCCCTTATGGTGCAGAAGAACAGATGAACTTGTTGATGGCCCAAACGCATCATATATTACCCCGG	4327521	4328953	splits:1:31:12:C:P	0.023
	canonical	PSY1-circ1	AGTTCTTTGATGAGGCAGAGGAAGGCGTGACAGAATTGAGCTCAGCTAGTAGATTCCCTCCATTCAGAGATATGATTGAAGGAATGCGTATGGACTTGAGA	4328007	4328953	splits:1:22:12:C:P	0.023
ACBR-2	canonical	PSY1-circ2	CCACCTATCTAAGGCTGCCGGGGTAATATATGATGCGTTTGGGCCATCAACAAGTTCATCTGTTCTTCTGCACCATAAGGGAATCTACTAGCTGAGCTCAA	4327521	4328953	splits:2:33:12:C:P	0.05
	non-canonical	PSY1-circ5	TATGAAGAAACAAATACATAGGGCAAGAAAGTTCTTTGGGCCATTTGACATGCTCGATGGTGCTTTGTCCGATACAGTTTCTAACTTTCCAGTTGATATTC	4327629	4328903	splits:1:1:1:C:P	0.025
	ciRNA		TACTGAGAACGACAAGCTCAATACAAGGTTATTTTGCCAGAATAGTAGACAAAGGTGATAAATAAGAGAACACTAAAGATACGAGGTATGGGATATTGTAA	4329384	4329616	splits:1:1:1:C:P	0.025
	canonical	PSY1-circ2	GGGCCATCAACAAGTTCATCTGTTCTTCTGCACCATAAGGGAATCTACTAGCTGAGCTCAATTCTGTCACGCCTTTCTCTGCCTCATCAAAGAACTTTCTT	4327521	4328953	splits:10:116:33:C:P	0.248
	canonical	PSY1-circ1	CAGAATTGAGCTCAGCTAGTAGATTCCCTCCATTCAGAGATATGATTGAAGGAATGCGTATGGACTTGAGAAAATCGAGATACAAAAACTTCGACGAACTA	4328007	4328953	splits:5:76:33:C:P	0.124
	non-canonical	PSY1-circ6	ATATTACCCCGGCAGCCTTAGATAGGTGGGAAAATAGGCTAGAAGATGTTTTCAATGGGCGGCCATTTGACATGCTCTACCTATTCCGGGGAATTTGGGCT	4326837	4327645	splits:2:2:2:C:P	0.05
	non-canonical	PSY1-circ7	CACTCATCAACCCAACCGTACCAGCAACATAATAACAATAAAGGACAAGAGATTTTGCATATGCAATAGGTAATGCAATCAACTTCTTTGATTTGCTCACA	4328082	4329784	splits:2:2:3:C:P	0.05

ACRed-1	non-canonical	PSY1-circ8	GTTTCTTCATAAAGATTCTCCATTTATCGGTCACCCTTCCAGCAAATATATCTTCATCGGATAGACCTGCCTCATCAAAGAACTTTCTTGCCCTATGTATT	4328811	4328912	splits:2:2:2:C:P	0.05
	canonical	PSY1-circ3	GCCATCAACAAGTTCATCTGTTCTTCTGCACCATATCTTCTCCAACATCTCTGAGTATGTTAGTTAATTGATTTGCGATCCCCAGAGCCAAAGCAGCATTA	4327521	4328242	splits:1:116:83:C:P	0.025
	non-canonical	PSY1-circ9	ACGAACTATACCTTTATTGTTATTATGTTGCTGGTACGGTTGGGTTGATGAGTGTTCCAATTATGGGTATCGCCCCTGAAATAGGCTAGAAGATGTTTTCA	4327600	4328148	splits:1:1:1:C:P	0.025
	ciRNA		AGCTCATTTTCCTATATTTTGATATGTAAATATAGCAAAAAAAATTATGTGCGTAAAACACAGCTTTGTACTTACAAAGAACAACGGAAGCCTAAATACGG	4328244	4328740	splits:1:1:1:C:P	0.025
ACRed-2	canonical	PSY1-circ2	GAAAGGCGTGACAGAATTGAGCTCAGCTAGTAGATTCCCTTATGGTGCAGAAGAACAGATGAACTTGTTGATGGCCCAAACGCATCATATATTACCCCGGC	4327521	4328953	splits:6:121:20:C:P	0.151
	non-canonical	PSY1-circ10	ATTGAAGCCAATGACTACAACAACTTCACAAAGAGAGCATATGTGAGCAAATCAAAGAAGTCTACTTGCCTCAAGATGAATTAGCACAGGCAGGTCTATCC	4328776	4329742	splits:4:4:4:C:P	0.101
	non-canonical	PSY1-circ11	CCACTCTCAAACAAAATTACCCACTTTCTTTTTGTCACCTTCCAAACTTTACTTAGTTTATAGAAAATAAATTACTCCTAGTAGATTCCCACTAGAGAATA	4326363	4326466	splits:2:2:2:C:P	0.05
	non-canonical	PSY1-circ12	AAGACGTTTAACTTAGGAACTATGCTAATGACTCCCGAGAGAAGAAGGGCTATCTGGGCAATATATGTATGGTGCAGAAGGCAGCCTTGGTGAAGAGGCAA	4326775	4327097	splits:2:2:117:C:P	0.05
	non-canonical	PSY1-circ13	CCGATAAATGGAGAATCTTTATGAAAGGGCTATCTGGGCAATATATGTATGGTGCAGAAGAACAGATGAACTTGTTGATGGCCCAAACGCATCATATATTA	4327075	4328870	splits:2:2:2:C:P	0.05
	non-canonical	PSY1-circ14	ACCCAACCGTACCAGCAACATAATAACAATAAAGGTATCTCAATTCTGTCACGCCTTTCTCTGCCTCATCAAAGAACTTTCTTGCCCTATGTATTTGTTTC	4328078	4328934	splits:1:1:1:C:P	0.025

Supplementary Table 4 Fruit color of PSY1-circ1 transgenic tomatoes (Ailsa Craig and microTom)

a. Fruit color of *PSY1-circ1* overexpression lines (AC, T0 generation)

Fruit color	OE lines	Total
Yellow	OE5, OE8, OE11, OE19, OE21, OE23, OE26	7
Red	OE3, OE4, OE7, OE12, OE13, OE14, OE15, OE28	8

b. Fruit color of *PSY1-circ1* overexpression lines (microTom, T0 generation)

Fruit color	OE lines	Total
Yellow	OE14, OE16,OE38,OE40,OE52,OE53	6
Red	OE1,OE9,OE11,OE15,OE17,OE18,OE19,OE22,OE23,OE24,OE27,OE28,OE29,OE30,OE35,OE36,OE41,OE43,OE44,OE45,OE50,OE51,OE55,OE56	24

c. Fruit color of *PSY1-circ1* overexpression lines (microTom, hygromycin-resistant T1 generation plants)

	OE lines	Yellow progenies	Red progenies
Yellow lines	OE16	12	0
	OE38	10	3
	OE53	8	0
Red lines	OE1	9	18
	OE9	0	7
	OE11	4	2
	OE18	0	15
	OE44	0	11

Supplementary Table 5　Primers used in this study

Name	Sequence (5’-3’)	Annotation
PSY1-cF1	GTTGGAGAAGATGCCAGAAG	Divergent primers for *PSY1-circ1*
PSY1-cR1	CCATACGCATTCCTTCAATC	
PSY1-lF1	TTGGTTTGCCTGTCTGTG	Convergent primers for *PSY1*
PSY1-lF2	TGTATCGGACAAAGCACC	
PDS-cF1	CTATGTCAAAGGCACTCAACT	Divergent primers for *PDS-circ1*
PDS-cR1	CCTAATTCTCCAAACAGGTTCT	
PDS-lF1	GACAGGGTGACAGATGAGGT	Convergent primers for *PDS*
PDS-lF2	CACAAAAGCATCTCCCTCG	
ZISO-cF1	TATGAGGATTACCAGGCATC	Divergent primers for *ZISO-circ1~2*
ZISO-cR1	CCATAACTGCACTCCATCGT	
ZISO-lF1	AAATGCCCACCAAAGAGC	Convergent primers for *ZISO*
ZISO-lR1	GTCAACAGCCGCTACCTC	
HP2-cF1	GATGGCTTCCTTGCCTTGT	Divergent primers for *HP2-circ1~4*
HP2-cR1	GGCGTGGATGTTGTTTGAG	
HP2-lF1	TTGGCAGTGTTGATGGTG	Convergent primers for *HP2*
HP2-lR1	GTTTGGAGGTGCTACGAAG	
PSY1-div-F	TCAGCTAGTAGATTCCCTCCATTC	Divergent primers for *PSY1-circ1* (for Real-time PCR)
PSY1-div-R	CCTTTGATTCAGGGGCGATAC	
PSY1-con-F	GGACAAGTTTCATGGAATCAGT	Convergent primers for *PSY1* (for Real-time PCR)
PSY1-con-R	TGGAGTAGCCAAAATAGCAGAC	
PDS-div-F	TGCTATGTCAAAGGCACTCAAC	Divergent primers for *PDS-circ1* (for Real-time PCR)
PDS-div-R	CATGCAGCTACCTGAAGAAACC	
PDS-con-F	GCCGGTGACTACACGAAACAG	Convergent primers for *PDS* (for Real-time PCR)
PDS-con-R	ACGCTTGCTTCCGACAACTTC	
HP2/1-div-F	CAGGTTTGACGAGAAGAGAGTGC	Divergent primers for *HP2-circ1,3,4* (for Real-time PCR)
HP2/1-div-R	TCAACAGGTGATGTCGGTCC	
HP2/2-div-F	TGATGGCTTCCTTGCCTTGT	Divergent primers for *HP2-circ2* (for Real-time PCR)
HP2/2-div-R	CAGCATTTCGGGATACCTTCTC	
HP2-con-F	AGCTATTGACCGCCATAGACAG	Convergent primers for *HP2* (for Real-time PCR)
HP2-con-R	GGGTGGAAGAGGAAGGAACA	
ETR4-cF1	CAAGGAGACATCTGGGTAATC	Divergent primers for *ETR4-circ1~2*
ETR4-cR1	GCACACAGTGAAACTCAAGC	
ETR4-lF1	TGAGCAGCGGCTTCTTGT	Convergent primers for *ETR4*
ETR4-lR1	GCACACAGTGAAACTCAAGC	
ACO6-cF1	CCAAAGTTAGCAACTACCCACC	Divergent primers for *ACO6-circ1~4*
ACO6-cR1	CTTGCCACCATTTCCTTG	
ACO6-lF1	ATGGTGGCAAGTAAAGGC	Convergent primers for *ACO6*
ACO6-lR1	AGTAGGACCCTTTGAGCC	
ZISO-div-F	ACTAGAGGTAGCGGCTGTTGAC	Divergent primers for

ZISO-div-R	GGGAAAGCATTACAATCTGTGG	*ZISO-circ1~2* (for Real-time PCR)
ZISO-con-F	ATGGGGACCGGAGGTTAGC	Convergent primers for *ZISO* (for Real-time PCR)
ZISO-con-R	GCATAATGGGGTGGAGGAAGTA	
SPL12-cF1	GGAAATGGGAGTGTTCAAAG	Divergent primers for *SPL12-circ1~2*
SPL12-cR1	CAGCCAATGTATGGTAACGAG	
SPL12-lF1	AGCAGGTTCCATAGCGTGTC	Convergent primers for *SPL12*
SPL12-lR1	CAGCCAATGTATGGTAACGAG	
Actin-con-F	GTCCTCTTCCAGCCATCCAT	Convergent primers for *actin* (for Real-time PCR)
Actin-con-R	ACCACTGAGCACAATGTTACCG	
F1	ATTTCTTGGTTGGTAGGGTG	Primers used for identification of circRNA and linear RNAs in PSY1-circ1 overexpression plants.
R1	TGCGAGGTTCATTTCTTGGT	
R2	ATCGACCTAGAAATACTCAACAC	
R3	CTGCCTGTGCTAATTCATCTTG	
cF1	GTTGGGTTGATGAGTGTTCC	
cR1	CAAGTCCATACGCATTCC	
PSY1-circ-v-lF	CATTTCTTGGTTGGTAGGGTG	Primers used for identification of linear RNAs generated from PSY1-circ1 overexpression vector. (for Real-time PCR)
PSY1-circ-v-lR	CTACAAACATTTCCGCTAGGCTG	
PDS-circ-v-lF	GAACTTATGGACAACCCAATCAC	Primers used for identification of linear RNAs generated from PDS-circ1 overexpression vector. (for Real-time PCR)
PDS-circ-v-lR	CGTAGAATCTACCAGAAAAGACAGC	
